# Context-dependent regulation of endothelial cell metabolism: differential effects of the PPARβ/δ agonist GW0742 and VEGF-A

**DOI:** 10.1038/s41598-020-63900-0

**Published:** 2020-05-12

**Authors:** Ashton Faulkner, Eleanor Lynam, Robert Purcell, Coleen Jones, Colleen Lopez, Mary Board, Kay-Dietrich Wagner, Nicole Wagner, Carolyn Carr, Caroline Wheeler-Jones

**Affiliations:** 1 Department of Comparative Biomedical Sciences, Royal Veterinary College, London, UK; 2 Present Address: Experimental Cardiovascular Medicine, Bristol Medical School, University of Bristol, Bristol, UK; 3 Department of Physiology Anatomy & Genetics, University of Oxford, Oxford, UK; 4 Université Côte d’Azur, Institute of Biology Valrose, Nice (iBV), CNRS UMR7277, INSERM U1091 Nice, France

**Keywords:** Cell migration, Cardiovascular biology, Angiogenesis

## Abstract

Peroxisome proliferator activated receptor β/δ (PPARβ/δ) has pro-angiogenic functions, but whether PPARβ/δ modulates endothelial cell metabolism to support the dynamic phenotype remains to be established. This study characterised the metabolic response of HUVEC to the PPARβ/δ agonist, GW0742, and compared these effects with those induced by VEGF-A. In HUVEC monolayers, flux analysis revealed that VEGF-A promoted glycolysis at the expense of fatty acid oxidation (FAO), whereas GW0742 reduced both glycolysis and FAO. Only VEGF-A stimulated HUVEC migration and proliferation whereas both GW0742 and VEGF-A promoted tubulogenesis. Studies using inhibitors of PPARβ/δ or sirtuin-1 showed that the tubulogenic effect of GW0742, but not VEGF-A, was PPARβ/δ- and sirtuin-1-dependent. HUVEC were reliant on glycolysis and FAO, and inhibition of either pathway disrupted cell growth and proliferation. VEGF-A was a potent inducer of glycolysis in tubulogenic HUVEC, while FAO was maintained. In contrast, GW0742-induced tubulogenesis was associated with enhanced FAO and a modest increase in glycolysis. These novel data reveal a context-dependent regulation of endothelial metabolism by GW0742, where metabolic activity is reduced in monolayers but enhanced during tubulogenesis. These findings expand our understanding of PPARβ/δ in the endothelium and support the targeting of PPARβ/δ in regulating EC behaviour and boosting tissue maintenance and repair.

## Introduction

Endothelial cells (ECs) lining normal vessels remain relatively quiescent, but retain the remarkable ability to acquire a highly active and dynamic phenotype following tissue injury or ischaemia^1^. This is essential for angiogenesis, during which ECs sprout, migrate and proliferate to establish a new vascular network. Cells undergoing such rapid and dynamic alterations in growth and proliferation are known to exhibit significant alterations in their metabolism^2^, so interest in targeting master metabolic regulators, or metabolic pathways directly, to influence EC behaviour is growing^3,4^. While the importance of various metabolic pathways for sustaining angiogenic behaviour is increasingly recognised^3,5^, knowledge of how ECs adapt their metabolism in response to extrinsic signals is particularly lacking.

Exposure to vascular endothelial growth factor A (VEGF-A), a well-studied angiogenic growth factor, is known to increase glycolytic flux in ECs, in part through stimulation of the phosphoinositide 3-kinase (PI3-K)/Akt signalling pathway and increased activity of key enzymes in the glycolytic cascade^6^. Characterisation of the metabolic response of ECs to other established and putative angiogenic factors, and the functional importance of these changes, has not been addressed to date. The peroxisome proliferator activated receptor β/δ (PPARβ/δ) nuclear receptor has been identified as a facilitator of the angiogenic response and synthetic PPARβ/δ agonists generally exhibit pro-angiogenic activity^7–10^. PPARβ/δ controls the expression of multiple genes linked to the regulation of glucose and lipid homeostasis that, alongside other metabolic regulators, directly impact on cell function^11–13^. However, knowledge of the metabolic effects of PPARβ/δ activation within the endothelium is currently limited and largely focused on the pathological microenvironment^14^. Thus, whether PPARβ/δ acts as a metabolic regulator within ECs to support the dynamic phenotype remains to be fully established.

The aim of this study was to characterise the metabolic response of ECs to the highly-selective PPARβ/δ agonist, GW0742^15^, and identify its relationship to EC dynamic behaviour. Accordingly, we show that GW0742 induces a context-dependent metabolic response by ECs, whereby metabolic activity is reduced in cell monolayers but elevated, in the form of enhanced fatty acid oxidation (FAO), during dynamic tubulogenesis. Furthermore, we identify a requirement for the metabolic co-regulator, sirtuin-1 (SIRT1), in supporting PPARβ/δ’s pro-tubulogenic effect. These findings increase our understanding of the role of PPARβ/δ within the endothelium and identify PPARβ/δ as a potential regulator governing the metabolic shift during angiogenesis.

## Results

### GW0742 and VEGF-A induce contrasting metabolic effects in HUVEC monolayers

**Figure 1:**
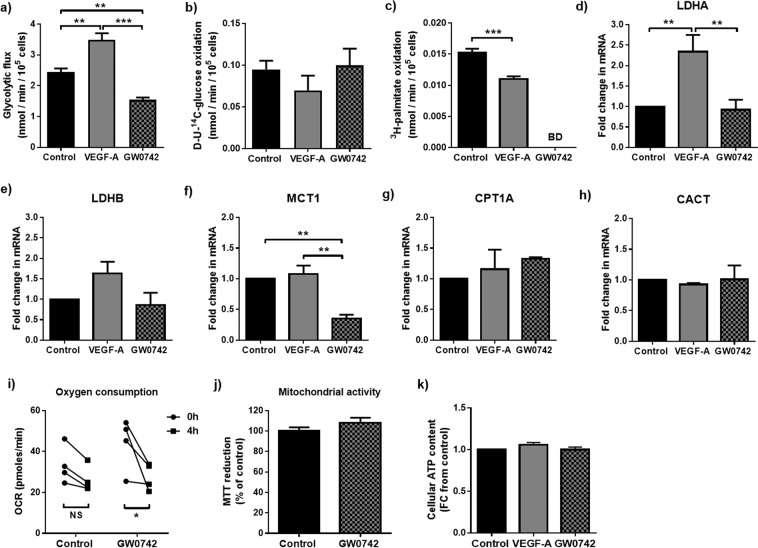
GW0742 reduces flux through central metabolic pathways whilst VEGF-A leads to metabolic activation in HUVEC monolayers. (**a**) GW0742 (100 nM; 4 h) significantly reduced, whilst VEGF-A (25 ng/ml; 4 h) significantly increased, glycolytic flux. **(b)** No change in D-U-^14^C-glucose oxidation rate was detected with either treatment. **(c)** Both VEGF-A (25 ng/ml; 5 h) and GW0742 (100 nM; 5 h) treatment reduced FAO. Data represent means (±S.E.M) of *n* = 3 to 5 ^**^p < 0.01 ***p < 0.001 BD = Below level of detection; as determined by one-way ANOVA followed by Tukey’s post-comparison test. (d - h) RT-qPCR showing changes in LDHA, LDHB, MCT1, CPT1A and CACT mRNA expression in HUVEC monolayers treated with VEGF-A (25 ng/ml; 4 h) or GW0742 (100 nM; 4 h). Data represent means (±S.E.M) of *n* = 3 to 5 **p < 0.01 as determined by one-way ANOVA followed by Tukey’s post-comparison test. (i) GW0742 (100 nM; 4 h) significantly reduces OCR. Data represent means (±S.E.M) of *n* = 4 *p < 0.05 as determined by two-way ANOVA followed by Bonferroni’s post-comparison test. Data were collected from 2 independent EC isolates across 2 repeats (j) GW0742 (100 nM; 4 h) has no effect on mitochondrial reducing potential. Data are means (±S.E.M) of *n* = 3. (k) Relative cellular ATP remains unchanged in HUVEC monolayers following treatment with VEGF-A (25 ng/ml; 4 h) or GW0742 (100 nM; 4 h). Data are means (±S.E.M) of *n* = 3.

Confluent HUVEC showed a preference for anaerobic glycolysis over that of glucose or FAO when cultured under standard conditions (Fig. 1a–c). As expected, HUVEC showed a significantly elevated rate of glycolytic flux following treatment with VEGF-A (25 ng/ml) compared with untreated controls, a response associated with increased lactate dehydrogenase A (LDHA) mRNA expression (Fig. 1a,d). A small but significant reduction in the rate of FAO (Fig. 1c) was also evident. However, expression of rate-governing enzymes involved in mitochondrial fatty acid uptake (carnitine palmitoyl transferase A (CPT1A) and carnitine acyl-carnitine translocase (CACT)) were unaffected (Fig. 1g,h). In contrast, treatment of confluent HUVEC with the PPARβ/δ ligand, GW0742 (100 nM), reduced glycolytic flux (Fig. 1a). This was accompanied by a decrease in mRNA expression of the lactate transporter, MCT1, but no change in expression of either of the two LDH isoforms (Fig. 1d–f). ^3^H_2_O production from ^3^H-palmitate fell below the reliable limit of detection in GW0742-treated cells (Fig. 1c), suggesting that treatment with GW0742 may reduce FAO in the contact-inhibited state. In line with this, Seahorse flux analysis showed that oxygen consumption rate (OCR) was significantly reduced in GW0742-treated cells (Fig. 1i), whereas CPT1A and CACT mRNA expression, as well as the overall reducing capacity of mitochondria (MTT reduction), were unchanged (Fig. 1g,h,j). No change in glucose oxidation rate (Fig. 1b) or cellular ATP content (Fig. 1k) was detected in either VEGF-A- or GW0742-stimulated cells.

### GW0742 but not VEGF-A-induced EC tubulogenesis is dependent on PPARβ/δ

**Figure 2:**
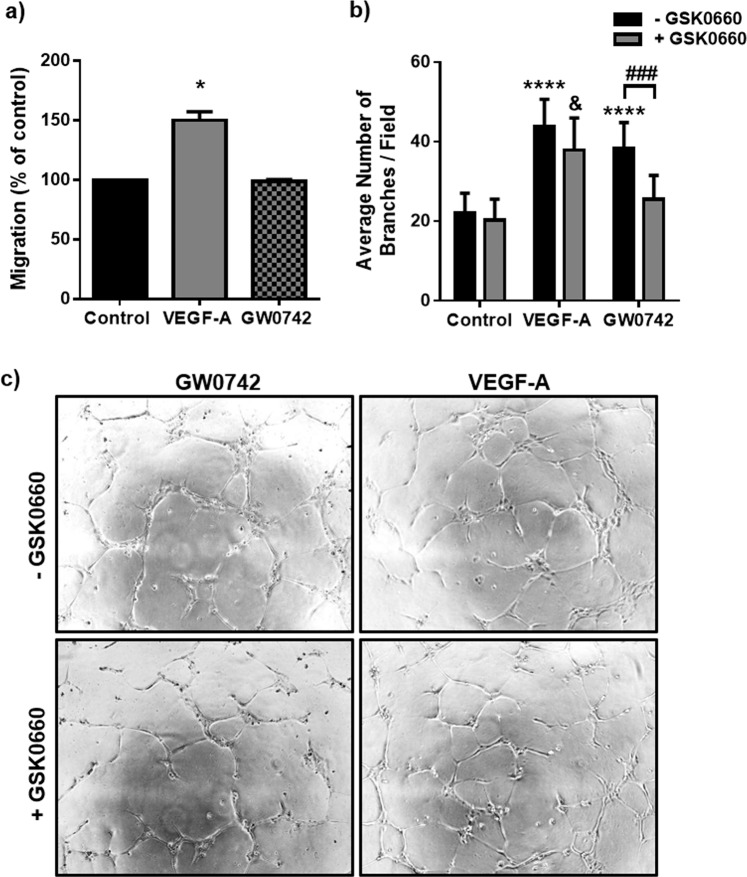
GW0742 induces HUVEC tubulogenesis in a PPARβ/δ-dependent manner. (**a**) VEGF-A (25 ng/ml), but not GW0742 (100 nM), significantly increased HUVEC migration in a scratch assay (16 h). Data represent mean (±S.E.M) % migration (*n* = 3); *p < 0.05 *vs*. untreated control as determined by one-way repeated measures ANOVA followed by Bonferroni’s post-comparison test. **(b)** GW0742 (100 nM), but not VEGF-A (25 ng/ml), induced capillary-like tube formation is significantly reduced in the presence of GSK0660 (1 µM) (*n* = 6). Data represent mean (±S.E.M) number of branches/field; ****p < 0.0001 *vs*. untreated control; ^###^*p* < 0.001; ^&^*p* < 0.05 *vs*. GSK0660 control; as determined by two-way repeated measures ANOVA followed by Bonferroni’s post-comparison test. **(c)** Representative images of capillary-like structures induced by GW0742 (100 nM) and VEGF-A (25 ng/ml) at 16 h in the absence or presence of GSK0660 (1 µM).

We next confirmed that agonist-activated PPARβ/δ stimulates EC dynamic behaviour *in vitro*. As GW0742 exerts some of its effects independently of PPARβ/δ^16^, the requirement for PPARβ/δ activation was confirmed using GSK0660, a PPARβ/δ antagonist. As shown in Fig. 2, incubation with GW0742 (100 nM) did not influence HUVEC migration, but significantly increased tubulogenesis in the tube formation assay. This response was abolished in the presence of a maximally effective concentration of GSK0660 (1 μM) (Fig. 2b and Supplementary Table S1), suggesting that the pro-tubulogenic effect of GW0742 was mediated through PPARβ/δ. As expected, VEGF-A (25 ng/ml) enhanced both EC migration and tube formation, with the latter being unaffected by treatment with GSK0660 (Fig. 2 and Supplementary Table S1), indicating that VEGF-A-induced HUVEC motility is not dependent on PPARβ/δ.

### VEGF-A and GW0742 exert differential forms of metabolic activation in motile HUVEC

**Figure 3:**
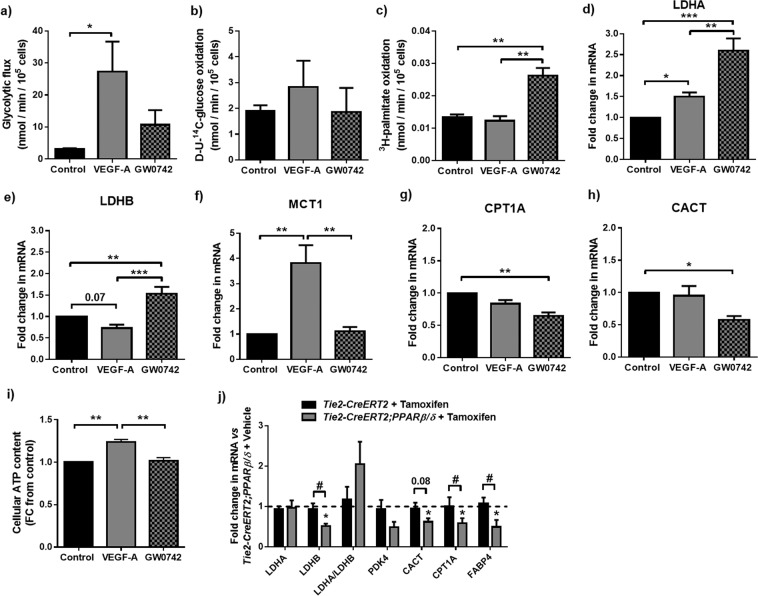
GW0742 and VEGF-A enhance flux through central metabolic pathways in dynamic HUVEC. (**a**) VEGF-A (25 ng/ml; 4 h), but not GW0742 (100 nM; 4 h), treatment significantly increased glycolytic flux. **(b)** No change in D-U-^14^C-glucose oxidation rate was detected with either treatment. **(c)** GW0742 (100 nM; 5 h), but not VEGF-A (25 ng/ml; 5 h), treatment significantly increased FAO. Data are means (±S.E.M) of *n* = 3–5; *p < 0.05 **p < 0.01 *vs*. untreated control as determined by one-way ANOVA followed by Tukey’s post-comparison test. **(d - h)** RT-qPCR showing changes in LDHA, LDHB, MCT1, CPT1A and CACT mRNA expression in tubulogenic HUVEC treated with VEGF-A (25 ng/ml; 4 h) or GW0742 (100 nM; 4 h). Data show means (±S.E.M) of *n* = 3–5 *p < 0.05 **p < 0.01 ***p < 0.001 as determined by one-way ANOVA followed by Tukey’s post-comparison test. **(i)** Relative cellular ATP level is significantly increased in sub-confluent HUVEC treated with VEGF-A but not GW0742. Data are means (±S.E.M) of *n* = 3 **p < 0.01 as determined by one-way ANOVA followed by Tukey’s post-comparison test. **(j)** RT-qPCR for metabolic genes in mouse cardiac ECs from control (*Tie2-CreERT2* + Tamoxifen; *n* = 5) or EC-selective tamoxifen-inducible PPARβ/δ overexpressing (*Tie2-CreERT2*;*PPARβ/δ*; *n* = 5) animals treated with tamoxifen compared to *Tie2-CreERT2*;*PPARβ/δ* animals (*n* = 5) treated with vehicle. Data are means (±S.E.M); *p < 0.05 *vs*. *Tie2-CreERT2*;*PPARβ/δ* + vehicle and ^#^*p* < 0.05 *vs*. *Tie2-CreERT2* + tamoxifen; as determined by one-way ANOVA followed by Tukey’s post-comparison test. Numbers above bars indicate *p*-values.

As in HUVEC monolayers, stimulation of tube formation with VEGF-A (25 ng/ml; 4 h) led to a 9-fold increase in glycolytic rate compared with untreated controls (Fig. 3a). In addition, a significant increase in LDHA and MCT1 gene expression was apparent at the time that mature EC networks were established (16 h) (Fig. 3d–f). Of note, despite the reduced rate of FAO in cell monolayers, FAO in VEGF-A-treated tube-forming cells was maintained (Fig. 3c), whilst CPT1A and CACT mRNAs were unaffected (Fig. 3g,h). On the other hand, stimulation of tube formation with GW0742 (100 nM) resulted in a significant 2-fold increase in FAO compared to untreated controls (Fig. 3c), whilst glycolytic flux was maintained (Fig. 3a). It is interesting to note that although GW0742 increased FAO, a significant up-regulation of LDHA mRNA expression was seen in established tubes (16 h) (Fig. 3d), while both CACT and CPT1A expression was significantly reduced (Fig. 3g,h). As observed in cell monolayers, glucose oxidation rate was unaffected by VEGF-A or GW0742 treatment (Fig. 3b). To determine if these metabolic changes influenced the level of ATP within dynamic cells, change in relative ATP content was assessed. It was not possible to assess ATP in the small number of cells used in the tube-formation assay, but a significant increase in ATP content was seen in active sub-confluent cells following treatment with VEGF-A but not with GW0742 (Fig. 3i).

### The transcriptional response observed in cardiac EC from PPARβ/δ overexpressing mice is similar to that elicited in HUVEC by GW0742

To identify whether PPARβ/δ-induced angiogenesis *in vivo* is associated with similar changes in EC metabolic phenotype, cardiac ECs from the previously-reported inducible conditional endothelial-specific mouse model of PPARβ/δ overexpression were utilised^17^. In this model, Cre-mediated PPARβ/δ overexpression is induced approximately 3-fold upon treatment with tamoxifen and leads to induction of an angiogenic programme within the heart^17^. RT-qPCR analysis of murine cardiac ECs immediately following their isolation revealed a similar reduction in mRNA expression of genes encoding enzymes involved in both glucose (LDHB) and lipid metabolism (CPT1A and CACT) in cells isolated from mice 1 week following treatment with tamoxifen (33 mg/kg/day), compared with animals treated with vehicle alone (Fig. 3j). These data suggest that in line with the role of PPARβ/δ identified in other tissues^11–13^, PPARβ/δ-induced angiogenic activity is associated with a shift in EC metabolic phenotype.

### An intact glycolytic network is of greater importance for EC tubulogenesis than mitochondrial-derived ATP production

Given the changes to glycolysis and mitochondrial metabolism seen in both GW0742- and VEGF-A-treated cells, we next assessed the importance of glycolytic flux and mitochondrial-derived ATP production for each agonist in promoting tubulogenic behaviour.

**Figure 4:**
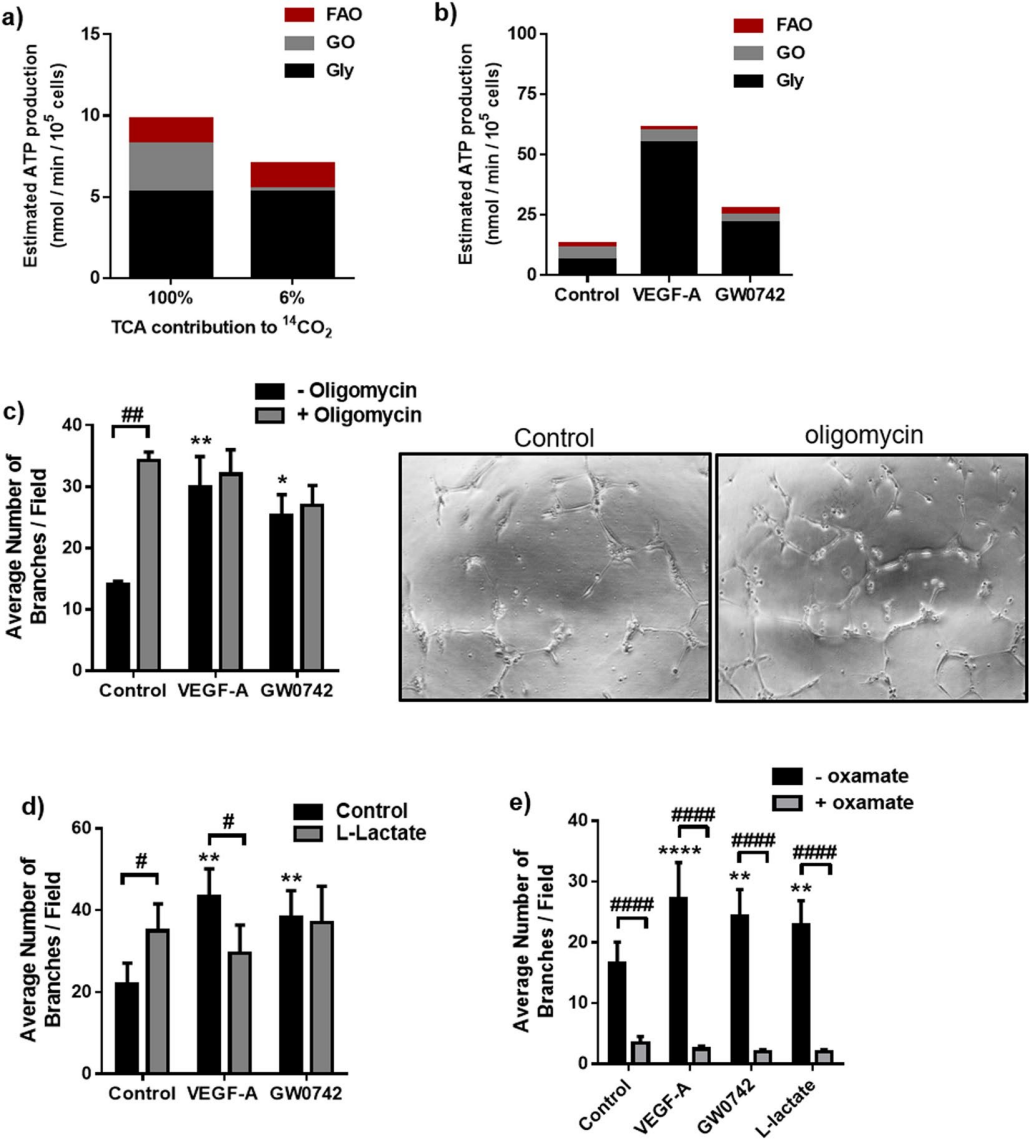
Mitochondrial ATP synthesis contributes less than glycolysis to HUVEC ATP production and is not essential for HUVEC tubulogenesis. (**a**) Glycolysis (Gly), compared with glucose oxidation (GO) and FAO, provides most of the estimated ATP under basal contact-inhibited conditions when assuming either 100% or 6% of the ^14^CO_2_ detected from D-U-^14^C-glucose metabolism arises from the TCA cycle. **(b)** Glycolysis, compared with glucose oxidation and FAO, is the largest contributor to estimated ATP production rate in dynamic HUVEC under basal, VEGF-A (25 ng/ml) and GW0742 (100 nM) treated conditions when assuming 6% of the ^14^CO_2_ detected from D-U-^14^C-glucose metabolism arises from the TCA cycle. **(c)** Inhibition of mitochondrial ATP synthase with oligomycin A (2 µM) lead to a significant increase in the number of capillary-like tubes formed by HUVEC at 16 h and had no significant effect on VEGF-A (25 ng/ml) or GW0742 (100 nM) induced tubulogenesis. Data are means (±S.E.M) number of branches/field from *n* = 3. *p < 0.05 **p < 0.01 vs. untreated control; ^##^*p* < 0.01; as determined by two-way repeated measures ANOVA followed by Bonferroni’s post-comparison test. (d) L-lactate (10 mM) significantly increased the number of capillary-like structures formed by HUVEC at 16 h and significantly reduced VEGF-A (25 ng/ml), but not GW0742 (100 nM)-induced tube formation. Data represent means (±S.E.M) of *n* = 6; **p < 0.01 *vs*. untreated co*n*trol; ^#^*p* < 0.05 ^##^*p* < 0.01; as determined by two-way repeated measures ANOVA followed by Bonferroni’s post-comparison test. (e) Direct inhibition of LDH with oxamate (1 mM) lead to significant reduction in capillary-like tube formation by HUVEC at 16 h under basal, VEGF-A (25 ng/ml), GW0742 (100 nM) and L-lactate (10 mM) conditions. Data represent mean (±S.E.M) number of branches/field from *n* = 5. **p < 0.01 ****p < 0.0001 *vs*. untreated control; ^####^*p* < 0.0001; as determined by two-way repeated measures ANOVA followed by Bonferroni’s post-comparison test.

First, using the metabolic flux data reported in Figs. 1 and 3, the potential contribution of mitochondrial oxidative phosphorylation (OXPHOS) and glycolysis to ATP production was calculated. In resting HUVEC, assuming that 100% of the ^14^CO_2_ detected from ^U-14^C-glucose metabolism arose from the TCA cycle, this would translate to <50% of calculated ATP production (from the three pathways tested) being supplied through mitochondrial OXPHOS (Fig. 4a). However, as studies show that <6% of measured ^14^CO_2_ metabolised by ECs arises from TCA cycle activity^18^, the contribution of mitochondrial OXPHOS to ATP production is likely to be even lower, with the majority (>70%) instead being supplied through anaerobic glycolysis (Fig. 4a). This is in line with that reported by others^3^. Even when cells were undergoing agonist-induced tubulogenesis, the estimated contribution of mitochondrial-derived ATP remained much lower than that supplied by glycolysis (Fig. 4b).

To directly assess the requirement for a functioning mitochondrial ATP-synthase in fuelling HUVEC dynamic activity, we made use of the ATP synthase inhibitor, oligomycin A. Unexpectedly, treatment with oligomycin A (2 μM; 16 h) led to a significant and robust induction of tube formation, comparable to that induced by the other two agonists (Fig. 4c and Supplementary Table S2). When oligomycin A was combined with VEGF-A, no inhibitory or additive effects were evident. However, when GW0742 and oligomycin A were combined, the number of tubes formed was still significantly greater than in control conditions, but other parameters measured, such as the number of junctions, were not (see Supplementary Table S2). These data indicate that ATP production through mitochondrial ATP synthase is not a fundamental requirement for supporting HUVEC dynamic behaviour *in vitro*, particularly in the presence of VEGF-A. However, impedance of mitochondrial ATP synthase activity may partially disrupt the actions of GW0742 in terms of connectivity of formed tubes but not overall tube number.

In contrast, when glycolytic flux was suppressed using the 6-phosphofructo-2-kinase/fructose-2,6-biphosphatase 3 (PFKFB3) inhibitor, 3PO (15 μM)^3,19^, HUVEC were unable to form capillary-like structures after stimulation with either VEGF-A (25 ng/ml) or GW0742 (100 nM) (see Supplementary Fig. S1), and a complete cytostatic effect was observed in the presence of the PFKFB3 inhibitor. However, HUVEC, in both the tube formation assay and in monolayers, were still capable of reducing MTT, indicating that a degree of mitochondrial function was retained under these conditions. Furthermore, in cell monolayers, exposure to 3PO (15 μM; 16 h) was associated with a 15% reduction in mitochondrial reducing potential (see Supplementary Fig. S1), suggesting that 3PO treatment may lead to an overall reduction in metabolic activity.

As an alternative means of reducing glycolytic flux, HUVEC were co-incubated with 10 mM exogenous L-lactate, a concentration sufficient to reduce glycolytic flux in HUVEC monolayers (see Supplementary Fig. S2). The presence of exogenous L-lactate led to a significant reduction in VEGF-A-induced tube formation but did not affect GW0742-induced tubulogenesis (Fig. 4d and Supplementary Fig. S2 & Table S3). Moreover, L-lactate alone induced dynamic tube formation. A reduced lactate concentration in the medium (from 10 to approximately 5 mM) at the end of the assay (16 h), suggests that this positive motile effect was associated with the direct uptake of L-lactate by HUVEC (see Supplementary Fig. S2). Although glucose oxidation was significantly reduced in cells undergoing L-lactate-induced tubulogenesis versus untreated control cells, interestingly the rate of glycolysis was maintained, and a cytostatic effect was still observed in the presence of the glycolysis inhibitor, 3PO (see Supplementary Fig. S2). When LDH was directly inhibited with oxamate (1 mM), a structural analogue of pyruvate, this reduced HUVEC tubulogenesis under basal, VEGF-A, GW0742, and L-lactate treated conditions (Fig. 4e and Supplementary Fig. S3 & Table S4), suggesting that an active LDH enzyme is essential for supporting HUVEC dynamic behaviour, independent of the stimulus.

### Mitochondrial FAO contributes to growth factor-induced EC growth and motility

Although not a major contributor to EC ATP production, a healthy and functional mitochondrial network is vital for efficient cell growth and function. In ECs, VEGF-A has been shown to stimulate mitochondrial biogenesis^20^ and since studies have demonstrated that PPARβ/δ can facilitate mitochondrial biogenesis in other cell types^21^, the effects of GW0742 and VEGF-A on mitochondrial biogenesis/dynamics were investigated.

Treatment of confluent HUVEC with VEGF-A (25 ng/ml; 4 h) was not associated with any significant change in mRNA expression of mitochondrial transcription factor A (TFAM), a transcription factor involved in mitochondrial biogenesis, or the mitochondrial fusion protein 2 (MFN2) (see Supplementary Fig. S4). However, VEGF-A treatment did result in a significant increase in relative mitochondrial DNA content (*p* = 0.02), as measured by qPCR, and a subtle change in mitochondrial morphology and number by visual inspection following incubation with MitoTracker Green (see Supplementary Fig. S4). No change in either TFAM mRNA expression, mitochondrial DNA content, or MitoTracker Green staining was detected following treatment with GW0742 (100 nM; 4 h) (see Supplementary Fig. S4). However, after VEGF-A and GW0742-induced tube formation (16 h), mitochondria appeared more aligned within the tubes and there was an increase in MFN2 mRNA expression (see Supplementary Fig. S4), suggesting a common change in mitochondrial tethering upon HUVEC tubulogenesis.

It has recently been suggested that mitochondrial FAO is optimised to uniquely supply carbon for the synthesis of nucleotides necessary for EC proliferation^5^. As an elevated rate of FAO was observed in GW0742-induced tubulogenic cells (see Fig. 3c), the effect of GW0742 treatment on HUVEC proliferation was assessed.

**Figure 5:**
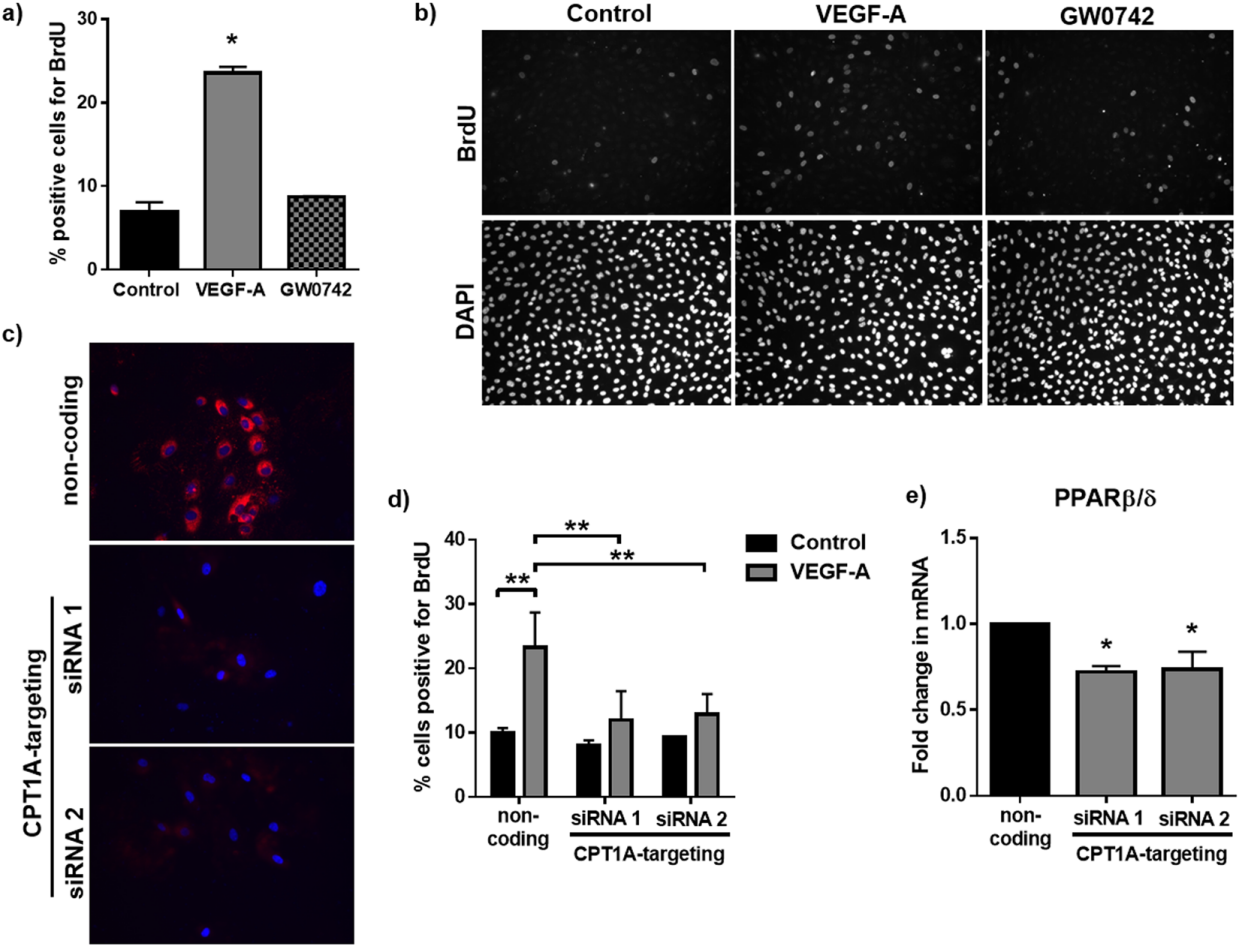
VEGF-A but not GW0742 promotes HUVEC proliferation which requires an intact FAO pathway. (**a**) VEGF-A (25 ng/ml) but not GW0742 (100 nM) significantly increased HUVEC proliferation (24 h), as assessed by BrdU incorporation. Data represent mean (±S.E.M) percentage of BrdU^+^ cells from *n* = 3; *p < 0.05 *vs*. untreated control as determined by one-way repeated measures ANOVA followed by Tukey’s post-comparison test. **(b)** Representative images showing BrdU incorporation following control, VEGF-A (25 ng/ml) or GW0742 (100 nM) treatment for 24 h. **(c)** Representative Immunofluorescence images showing a significant reduction in CPT1A expression 48 h following transfection with CPT1A-targeting siRNA sequences (10 nM). **(d)** VEGF-A-induced (25 ng/ml; 24 h) proliferation (BrdU incorporation) was significantly reduced in CPT1A-silenced HUVEC. Data represent means (±S.E.M) of *n* = 3. **p < 0.01 as determined by two-way ANOVA followed by Bonferroni’s post-comparison test. **(e)** PPARβ/δ mRNA expression is significantly reduced in CPT1A-silenced cells 48 h post-transfection (*n* = 3). Data represent means (±S.E.M); *p < 0.05 *vs*. non-coding transfection control as determined by one-way ANOVA followed by Bonferroni’s post-comparison test.

As expected, VEGF-A (25 ng/ml) significantly enhanced HUVEC proliferation at 24 h, as assessed by BrdU incorporation (Fig. 5a,b) and nuclear counting (Supplementary Fig. S5), whereas treatment with GW0742 (100 nM) had no effect. To understand the importance of mitochondrial FAO for VEGF-A-induced HUVEC proliferation, siRNA was used to achieve targeted knock-down of CPT1A. Immunofluorescence imaging showed effective silencing of CPT1A was achieved at a siRNA concentration of 10 nM using two independent siRNAs (Fig. 5c). This was also confirmed by RT-qPCR (for siRNA 1 & 2) and western blot (for siRNA 1 only) (Supplementary Fig. S5). The increase in HUVEC proliferation in response to VEGF-A (25 ng/ml; 24 h) was still evident in cells transfected with non-coding siRNA (10 nM), as assessed by BrdU incorporation, but was abolished in CPT1A-silenced HUVEC (Fig. 5d and Supplementary Fig. S5), supporting a role for FAO in VEGF-A-induced HUVEC proliferation.

As the PPARβ/δ agonist did not affect HUVEC proliferation, the importance of mitochondrial FAO for HUVEC growth into capillary-like branches was investigated. Although siRNA against CPT1A successfully reduced CPT1A expression, PPARβ/δ mRNA expression was also significantly down-regulated in cells where CPT1A had been silenced (Fig. 5e). This effect was observed following silencing with both CPT1A-targeting siRNAs, suggesting an intricate relationship between CPT1A-governed FAO and PPARβ/δ expression, making it difficult to decipher if any functional effect on GW0742-induced tubulogenesis reflects a reduction in CPT1A activity or the associated reduction in PPARβ/δ expression.

To avoid the problem with CPT1A silencing, we then used a pharmacological approach employing the generic CPT1 inhibitor, etomoxir (ETO). ETO is widely used at concentrations of 40 μM or higher^4^. However, the presence of 40 μM ETO in the tube-formation assay led to significant cell death, as indicated by the detachment of cells from the substratum. In subsequent investigations using ETO at 1 and 10 μM, a varied response by individual HUVEC isolates was observed. In some cases, the presence of ETO at either concentration led to significant cell detachment from the matrix, whilst in other isolates, ETO had no effect on basal tube-formation but reduced the number of tube-like branches formed under both agonist-treated conditions after 16 h (see Supplementary Fig. S6).

Although it was not possible to reliably confirm a role for FAO in agonist-induced tubulogenesis, these data suggest that disruption of FAO for a prolonged period can significantly disrupt EC growth and proliferation.

### GW0742 but not VEGF-A-induced tubulogenesis is dependent on the metabolic co-regulator SIRT1

PPARβ/δ-mediated effects have been reported to be SIRT1-dependent^22^, and SIRT1 itself has recently been linked to the metabolic regulation of angiogenesis through a mechanism involving deacetylation of the FOXO1 transcription factor^23–25^. This prompted us to investigate the role of the SIRT1/FOXO1 axis in facilitating GW0742-induced tube formation.

**Figure 6:**
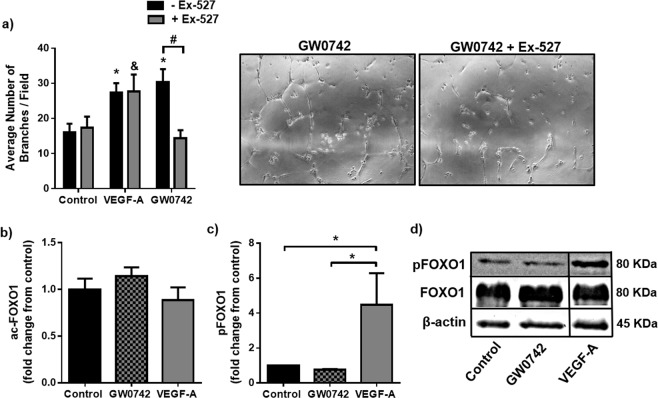
GW0742-induced HUVEC tubulogenesis is SIRT1-dependent, but independent of any change in FOXO1 activation status (**a**) GW0742 (100 nM), but not VEGF-A (25 ng/ml), mediated tube formation was significantly reduced in the presence of the selective SIRT1 inhibitor, Ex-527 (1 µM), at 16 h. Data represent mean (±S.E.M) number of branches/field from *n* = 3. *p < 0.05 *vs*. untreated control; ^#^*p* < 0.05; ^&^*p* < 0.05 *vs*. Ex-527 control; as determined by two-way repeated measures ANOVA followed by Bonferroni’s post-comparison test. **(b)** FOXO1 acetylation status was not changed following treatment (1 h) with either VEGF-A (25 ng/ml) or GW0742 (100 nM). Data represents means (±S.E.M) from *n* = 3. For each *n* number, fluorescence intensity was analysed from 150 cells per treatment condition. **(c)** Densitometry analysis showing that FOXO1 phosphorylation is not significantly changed following treatment (1 h) with GW0742 (100 nM) (*n* = 3) but is significantly increased following treatment (1 h) with VEGF-A (25 ng/ml) (*n* = 2) compared to untreated control (*n* = 3). Data represent means (±S.E.M). *p < 0.05 *vs*. untreated control as determined by one-way ANOVA followed by Bonferroni’s post-comparison test. **(d)** Representative western blot showing phosphorylated FOXO1, total FOXO1 and β-actin loading control. Boxed areas indicate non-adjacent lanes on the original blot (see Supplementary Fig. S8).

As indicated in Supplementary Fig. S6, immunofluorescence staining showed that SIRT1 was predominantly located within the nuclear and perinuclear region of HUVEC when cultured as contact-inhibited monolayers. Importantly, SIRT1 maintained its nuclear location upon stimulation to form tube-like structures with either GW0742 (100 nM) or VEGF-A (25 ng/ml). No significant increase in SIRT1 mRNA expression was observed under any treatment condition, either in HUVEC monolayers or following tubulogenesis (see Supplementary Fig. S7). When SIRT1 activity was inhibited using the highly-selective inhibitor, Ex-527 (1 μM), GW0742-induced tube formation was significantly reduced, whereas basal and VEGF-A-driven tubulogenesis were unaffected (Fig. 6a and Supplementary Table S5).

To identify whether the SIRT1-dependency of GW0742’s effect on tube formation was due to the requirement for SIRT1-mediated regulation of FOXO1, the acetylation status of FOXO1 was investigated. Immunofluorescence microscopy using an antibody specific for acetylated FOXO1 (acFOXO1) showed that the acetylated form of this transcription factor was almost exclusively located within the nuclear compartment of HUVEC and that the degree of acetylation was not influenced by either GW0742 (100 nM; 1 h) or VEGF-A (25 ng/ml; 1 h) (Fig. 6b and Supplementary Fig. S7). As VEGF-A is known to rapidly (within 1 h post-stimulation) inactivate FOXO1 through Akt-mediated phosphorylation^26,27^, the phosphorylation status of FOXO1 (pFOXO1) was measured in parallel. As expected, treatment of confluent HUVEC with VEGF-A (25 ng/ml; 1 h) led to an increase in FOXO1 phosphorylation, as assessed by western blotting, while incubation with GW0742 (100 nM; 1 h) did not affect FOXO1 phosphorylation status (Fig. 6c,d).

Together these data show that SIRT1 is predominantly located within the nucleus of endothelial cells and that whilst VEGF-A leads to FOXO1 inactivation and SIRT1-independent HUVEC tubulogenesis, GW0742-induced HUVEC tubulogenesis is a SIRT1-dependent process but independent of any significant change in FOXO1 status.

## Discussion

The importance of metabolism for regulating angiogenic behaviour is increasingly recognised^3,5^ but knowledge of how ECs adapt their metabolism in response to extrinsic signals is still lacking. Although a pro-angiogenic role for the PPARβ/δ nuclear receptor has been demonstrated^7,10,17^, whether this effect is mediated through alterations in EC metabolism has not been thoroughly investigated. We therefore aimed to compare the functional and metabolic responses of HUVEC treated with the pharmacological PPARβ/δ agonist, GW0742, with that of VEGF-A which has well-established proliferative, migratory and tubulogenic effects^28^.

The results presented here suggest that unlike VEGF-A, treatment of HUVEC with a selective concentration of GW0742 (100 nM)^15^ is not sufficient to induce short-term proliferation or migration. In line with this, it has been reported that GW0742 fails to induce proliferation of human retinal microvascular ECs at 24 h^10^, and similar results have also been described with the alternative PPARβ/δ agonist, GW501516^29,30^. This contrasts with findings reported by others demonstrating pro-proliferative and pro-migratory effects of PPARβ/δ agonists on ECs^7,31,32^. These discrepancies may reflect the time-point analysed, as the pro-proliferative effects were principally observed following long-term treatment (3–14 days) as opposed to the 24 h time point analysed here.

The contrasting functional responses of HUVEC to GW0742 and VEGF-A stimulation may be explained in part through the differential metabolic response induced by these two agonists. VEGF-A activates multiple signalling pathways that can lead to an increased rate of glycolysis and induction of mitochondrial biogenesis in preparation for cell proliferation^3,20^, a response also observed in the present study in HUVEC monolayers. Increased glycolytic flux can support migratory behaviour as many glycolytic enzymes are located alongside cytoskeletal proteins, providing hotspots of localised ATP production^3^. A high level of glycolysis also supports proliferation, as flux through glycolysis and its associated side-pathways provides the carbon necessary for the generation of new biomass^33,34^.

In contrast, treatment of HUVEC monolayers with GW0742 caused a reduction in glycolysis, FAO and oxygen consumption, indicating that PPARβ/δ may instead facilitate a form of metabolic quiescence. Importantly, this response was not associated with a compromise in overall mitochondrial activity, as no changes in mitochondrial DNA content, MitoTracker staining or MTT reducing capacity were observed, and cellular ATP content remained stable. This phenotype could, in part, contribute to the known anti-inflammatory and cyto-protective effects of PPARβ/δ agonists within the vasculature^35–38^, as a less active metabolism could be a mechanism by which ECs afford further protection from unnecessary oxidative stress arising from mitochondrial ROS production^39^. Furthermore, lowered EC metabolic rate could optimise nutrient transfer through the endothelium, thereby facilitating the delivery of nutrients to underlying tissues, as has been indicated in the heart^40^. It is tempting to speculate that these metabolic effects may contribute to the perceived benefits exerted by PPARβ/δ agonists on reperfusion-induced injury^41–43^. Interestingly, the metabolic response following GW0742 treatment demonstrated here is similar to that described for other vascular protective stimuli^25,44^, suggesting that this may be a common cyto-protective mechanism.

Whilst migration and proliferation were unaffected by GW0742 treatment, both GW0742 and VEGF-A were able to exert a tubulogenic effect. Similar pro-angiogenic responses to PPARβ/δ activation have been reported previously^7–10,17^. Furthermore, we now show that this tubulogenic effect is dependent on the deacetylase enzyme and metabolic co-regulator, SIRT1. SIRT1 signalling has previously been linked with regulating mitochondrial FAO^45^, as well as co-ordinating angiogenic activity, with SIRT1-silenced HUVEC showing a reduction in PDGF-β expression^23,24^, a key mediator in the recruitment of mural cells necessary for vessel stabilisation^46^. Given the lack of effect of the PPARβ/δ agonist on EC migration and proliferation, it seems reasonable to propose that PPARβ/δ may co-operate alongside SIRT1 within the nuclear compartment to regulate neo-vessel maturation/stabilisation.

It is possible that several SIRT1 target proteins are involved in facilitating the tubulogenic response^23,24,47^. One such target is the FOXO1 transcription factor which also plays a vital role in the regulation of EC metabolism and in the maturation/stabilisation phase of angiogenesis^25^. However, the absence of any significant change in acetylation or phosphorylation with GW0742 treatment suggests that FOXO1 is unlikely to be a major target in this instance. Given that only a short time-point was investigated, involvement of FOXO1 at a later time-point cannot be ruled out.

Vessel maturation may also be supported through alterations in mitochondrial FAO. FAO has been shown to be required for the establishment of stable networks on Matrigel^TM^ through facilitating EC-EC contact and regulating permeability^4^. In keeping with this, SIRT1-dependent GW0742-induced tubulogenesis was associated with an increased rate of FAO, a response not seen with exposure to VEGF-A. Unexpectedly, however, by 16 h of tubulogenesis in the presence of GW0742, both CACT and CPT1A genes had become down-regulated. This profile was also evident in cardiac ECs isolated from mice with selective endothelial PPARβ/δ overexpression, a model characterised by enhanced angiogenesis within the heart^17^. Although we cannot exclude that mouse cardiac ECs may have a slightly different metabolic response to that of HUVEC, the data for both cell types are in close agreement with each other. As such, this down-regulation of gene expression at the latter stages of both GW0742-induced HUVEC tubulogenesis *in vitro* and PPARβ/δ-induced angiogenesis *in vivo* may indicate a regulatory feedback loop designed to prevent a persistently elevated rate of FAO that could lead to a disturbance in local metabolic homeostasis. Indeed, rather than the physiological cardiac hypertrophy observed with the PPARβ/δ agonist, endothelial-specific PPARβ/δ overexpressing mice develop a pathological cardiac hypertrophy that was suggested to be a consequence of an altered balance of PPARβ/δ activity between the vascular and muscular compartments^8,17^.

Although an induction of FAO by PPARβ/δ activation has been established, its functional role remains unclear. Despite respiration in ECs being highly coupled with ATP synthesis^48^, increasing FAO to fuel mitochondrial-derived ATP does not appear to be a primary factor, as incubation with oligomycin, an inhibitor of mitochondrial ATP synthase, did not significantly impair the ability of HUVEC to form tube-like structures. This is in line with the fact that ECs principally obtain their ATP through glycolysis^3^. Moreover, an increase in tube-formation at 16 h of oligomycin treatment was evident, likely because of a compensatory increase in glycolytic flux that is known to occur following ATP synthase inhibition^48^. When coupled with the basal level of stimulation provided by the matrix, this adaptation may be enough to induce HUVEC tubulogenesis *in vitro*. Reports also present the possibility of oligomycin-induced VEGF production^49^. However, this effect was described in a monocytic cell line and further investigation would be required to determine whether similar mechanisms are operative in ECs. Beyond ATP production, fatty acids offer a rich source of carbon beneficial for generating biomass. ECs appear to be one of only a few cell types to utilise FAO for supplying carbon to support nucleotide synthesis vital for proliferation^5^. Although this role for FAO has yet to be confirmed by others, in support of such a concept, the VEGF-A-induced proliferation reported in the current study was completely abolished in CPT1A-silenced HUVEC. However, it is unlikely that the increase in FAO following GW0742 treatment is used to fuel proliferation, as proliferative activity was not affected by GW0742 treatment (as discussed above), and would not be expected to play a significant role over the time-frame of the tube-formation assay (16 h).

Understanding the role of FAO in facilitating GW0742-induced tubulogenesis was made challenging by the tight relationship that exists between the PPARβ/δ signalling pathway and FAO. Variable responses by HUVEC to the generic CPT inhibitor, ETO, made the pharmacological approach unreliable. However, when attempts were made to silence CPT1A, this led to a concomitant reduction in PPARβ/δ gene expression. Despite attempts to measure PPARβ/δ protein, by both western blotting and immunofluorescence analysis, the lack of reliable, commercially available PPARβ/δ-selective antibodies^50^ meant that protein levels could not be reliably assessed. Despite not being able to perform tube-formation assays in CPT1A-silenced cells, it is tempting to suggest that such a reciprocal regulatory relationship between PPARβ/δ and CPT1A is an integral part of GW0742-induced tubulogenesis, where CPT1A-driven FAO influences PPARβ/δ transcriptional activity and possibly PPARβ/δ expression itself. Indeed, a similar association has been described in lymphatic ECs for the transcription factor PROX1^51^. CPT1A is a PROX1 target and CPT1A-driven FAO provides acetyl-CoA that can be utilised for acetylation-mediated epigenetic modifications that facilitate the transcriptional activity of PROX1 and its target genes^51^. Any such molecular interaction with PPARβ/δ, however, requires further investigation.

It is important to note that the increase in FAO by GW0742 is not at the expense of glucose metabolism, as glucose oxidation and glycolysis were maintained, the latter even slightly increasing, thereby contrasting with the response in EC monolayers. The advantage that glycolysis holds for facilitating motile behaviour has been outlined above and its importance is highlighted by the cytostatic effect observed following inhibition of glycolysis with the PFKFB3 inhibitor, 3PO. This effect was independent of the stimulus used and is identical to the response observed by Schoors *et al*.^19^. Moreover, the reliance in particular on anaerobic glycolysis (even in the presence of sufficient oxygen) to support HUVEC growth and motility was indicated by the up-regulation of LDHA mRNA in both VEGF-A- and GW0742-stimulated cells and by the global impairment in tubulogenesis in the presence of the LDH inhibitor, oxamate. This requirement for LDH-facilitated anaerobic glycolysis has previously been described in pulmonary microvascular ECs *in vitro* and *in vivo*^52,53^. Although a baseline level of LDH-facilitated anaerobic glycolysis is essential, the extent to which it requires up-regulating to support tubulogenic activity is agonist-dependent, as maximal tubulogenesis induced by VEGF-A, which was also the largest inducer of glycolytic flux, but not GW0742, was impaired when anaerobic glycolysis was maintained towards baseline levels through supplementing the culture medium with L-lactate. It is also worth noting that L-lactate alone exerted a pro-tubulogenic response, an effect also demonstrated by others^54^.

**Figure 7:**
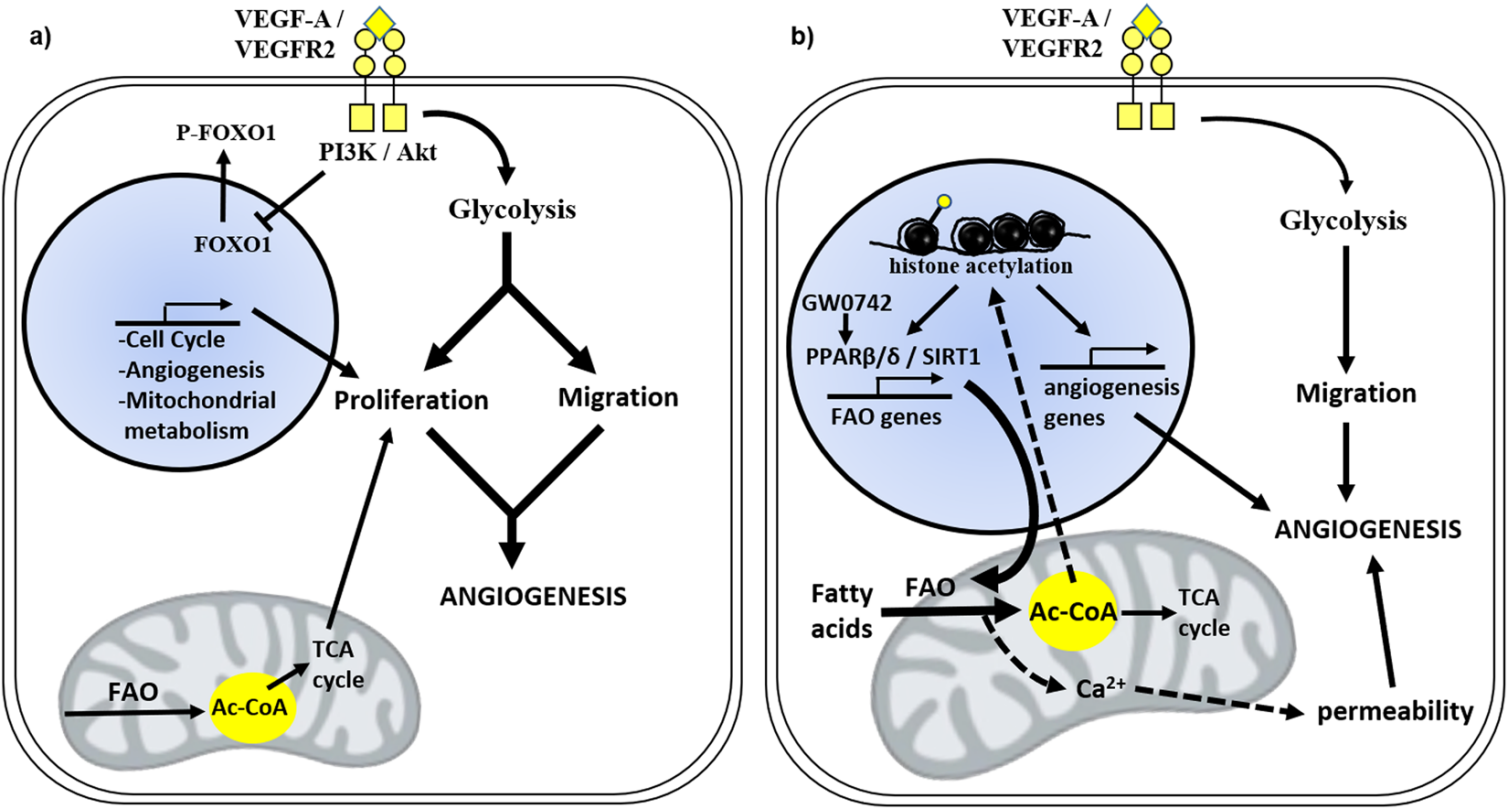
Schematic summary of VEGF-A- and GW0742-induced metabolic activation in dynamic HUVEC. (**a**) Treatment with VEGF-A is characterised by a significantly high rate of glycolysis which provides ATP for migration and activation of biosynthetic side-pathways for proliferation. At the same time, mitochondrial fatty acid oxidation (FAO) is maintained, which may further support proliferation through acting as an additional source of carbon. Key metabolites can also influence angiogenesis-related signalling pathways while Akt, through inhibitory phosphorylation, can inactivate the anti-proliferative transcription factor, FOXO1. **(b)** GW0742-induced tubulogenesis is SIRT1-dependent and characterised by a significant increase in mitochondrial FAO in addition to a moderate increase in glycolysis. Elevated acetyl-CoA levels may act as a substrate for post-translational protein acetylation (indicated in the figure by ) which could facilitate PPARβ/δ transcriptional activity and influence angiogenesis-related signalling pathways. FAO may also contribute to the regulation of EC permeability and thus may have a role in neovessel maturation/stabilisation. Thickness of arrows indicate increased flux while broken arrows indicate pathways that remain to be established.

Together, these findings expand our knowledge of the role of PPARβ/δ within the endothelium by identifying a context-dependent regulation of EC metabolism *in vitro* by GW0742. In contrast to the glycolytic activation observed in VEGF-A-stimulated EC monolayers and dynamic cells (Fig. 7a), GW0742-activated PPARβ/δ promotes a degree of metabolic quiescence in cell monolayers, but metabolic activation, in the form of elevated mitochondrial FAO, in tubulogenic cells (Fig. 7b). These actions are dependent on the metabolic co-regulator SIRT1. Further studies are now needed to decipher the specific interaction between these two metabolic regulators, as well as the functional role of GW0742-induced FAO within the angiogenic process, with a view to exploit these findings for therapeutic intervention.

## Methods

The data sets generated during the current study are available from the corresponding author on reasonable request.

### Cell isolation and culture

Human umbilical cord collection (obtained with informed written consent) and use of human endothelial cells conformed to the principles outlined in the Declaration of Helsinki and is approved by the NHS Health Research Authority East of England-Cambridge South Research Ethics Committee (REC reference 16/EE/0396). All experiments were performed in accordance with relevant guidelines and regulations. Human umbilical vein endothelial cells (HUVEC) were isolated and cultured as described previously and were used between passage 2 and 4^55^. Cells were cultured on 1% gelatin and maintained in M199 growth medium (M199 containing endothelial cell growth factor (20 μg/ml) and 20% (v/v) FBS). Experiments were performed in HEPES-buffered M199 containing 5mM L-glucose, 5mM L-glutamine, 1% FBS and 1% penicillin/streptomycin.

Endothelial cells were isolated from mouse hearts and gene expression analysed by reverse transcription quantitative PCR (RT-qPCR). All animal work was conducted according to national and international guidelines and was approved by the local ethics committee (Comité Institutionnel d‘Éthique Pour l’Animal de Laboratoire Azur, agreement number: PEA-NCE/2013/106). Age- and sex-matched tamoxifen-inducible Cre-mediated vascular-specific PPARβ/δ overexpressing animals (Tie2 CreERT2;PPAR*β*/*δ*) were injected for one week intraperitoneally either with sunflower oil (vehicle) or tamoxifen dissolved in sunflower oil (33 mg/kg per day), as previously described^17^. Tie2-CreERT2 animals injected with tamoxifen served as an additional control. Following terminal anaesthesia, hearts were excised, and cardiac endothelial cells were isolated by magnetic-activated cell sorting (MACS) using anti-CD31 beads (Miltenyi Biotec) prior to analysis by RT-qPCR.

### Tube formation assay

Tubulogenesis was assessed using the thin-layer angiogenesis (TLA) assay described previously^55^. Briefly, 5 μl/cm^2^ of Geltrex^TM^ basement matrix (ThermoFisher Scientific; UK) was spread evenly on to wells of 24- or 96-well plates as appropriate. HUVEC were seeded (25,000 cells/cm^2^) in technical duplicate (24-well plate) or sextuplet (96-well plate) and incubated for 16 h in the presence or absence of compounds of interest. Phase-contrast images (24-well plate: 4 images/well; 96-well plate: 1 image/well) were acquired using a Leica DMIRB inverted microscope (10x objective (NA = 0.25)) controlled through Axiovision software version 4.8.2 (Carl Zeiss Ltd; UK). For analysis, the mean number of branches per field was counted manually using ImageJ software.

### Scratch wound migration

Scratch wound closure was assessed as previously described^56^. Confluent HUVEC monolayers on gelatin-coated 24-well plates in M199 growth medium (5% FBS) were wounded and non-adherent cells removed by washing in PBS followed by addition of fresh experimental medium containing compounds of interest as detailed in the figure legends. Phase-contrast images were obtained at time 0 and 16 h (1 image/well) using a Leica DMIRB inverted microscope (10x objective (NA = 0.25)) controlled through Axiovision software version 4.8.2. Start and end gap distances were measured in pixels using ImageJ software and total migration after 16 h expressed as a percentage of control.

### Cell proliferation

HUVEC proliferation was quantified by nuclear staining (see Supplementary Methods) and by measuring 5-Bromo-2′-deoxyuridine (BrdU) incorporation. For BrdU incorporation experiments, HUVEC were seeded in triplicate on gelatin-coated 96-well plates (7,000 cells/well) in experimental medium supplemented with 10% FBS. The following day, cells were incubated with 10 µM BrdU (Sigma-Aldrich, UK) and compounds of interest for 4 hours in experimental medium. Cells were then fixed in 4% PFA for 10 min. Incorporated BrdU was detected by immunofluorescence using an anti-BrdU antibody (BioLegend, USA) and DAPI nuclear stain. Cells were imaged using a Leica DMIRB inverted microscope (20x objective). BrdU and DAPI positive nuclei were counted using the Analyze Particles function on ImageJ software. BrdU incorporation is given as the percentage of DAPI-positive nuclei which were BrdU-positive.

### Small interfering RNA (siRNA)

HUVEC were transfected in Opti-MEM transfection medium containing carnitine palmitoyltransferase-1A (CPT1A) targeting siRNAs (10 nM) obtained from Dharmacon/GE healthcare (ON-TARGET plus SMART pool) (siRNA 1) or Sigma Aldrich (siRNA ID: SASI_Hs01_00231321) (siRNA 2), pre-complexed with Lipofectamine RNAiMAX lipid carrier (ThermoFisher Scientific; UK) according to the manufacturer’s instructions. For all experiments, control wells/dishes containing cells transfected with non-coding control pool (10 nM) (Dharmacon; UK) were run in parallel. Plates were incubated for 24 h (37 °C/5% CO_2_) before replacing the transfection medium with M199 growth medium. Cells were used for experiments 48 h post-transfection. Knockdown was assessed by RT-qPCR, immunocytochemistry and western blotting.

### Reverse transcription-quantitative PCR (RT-qPCR)

Total RNA was extracted from cells using the Qiagen RNeasy Plus Mini Kit according to the manufacturer’s instructions. Complementary DNA (cDNA) was synthesised by the reverse transcription (RT) of mRNA using Oligo(dT) priming and SuperScript^TM^ II Reverse Transcriptase (ThermoFisher Scientific; UK). Quantitative PCR (qPCR), using both intercalator (SYBR^®^ Premix Ex Taq™; Clontech: TaKara Bio) and probe-based (TaqMan^TM^) technologies, was performed to determine changes in gene expression. CPT1A (Hs00912671_m1) and PPARβ/δ (Hs00606407_m1) TaqMan^TM^ primer-probes were obtained from Applied Biosystems (ThermoFisher Scientific; UK). All other primer sequences were designed using Primer3 and NCBI primer-BLAST software or obtained from published literature (see Supplementary Table S1). Minus cDNA template and H_2_O only controls were included for each gene of interest per experiment. Analysis was performed using the 2^−ΔΔCt^ method with results normalised to β-actin or TATA-box binding protein (TBP) internal control gene.

### Western blotting

Cells were lysed in RIPA buffer, supplemented with SigmaFast^TM^ protease and phosphatase inhibitors (Sigma Aldrich; UK), and protein concentration was determined by the Pierce BCA Protein Assay (ThermoFisher Scientific; UK). Protein samples (30 µg) were separated by SDS-PAGE at 150 V on 10% Tris-Glycine mini gels (ThermoFisher Scientific, UK) for 60–90 min. Resolved proteins were transferred to Hybond^TM^ polyvinylidene difluoride (PVDF) membrane (0.45 µm; GE Healthcare; UK) by semi-dry transfer (0.8 mAmps/cm^2^). Membranes were blocked (5% milk or 2.5% BSA; 1 h) and probed with primary antibodies at 4 °C overnight (anti-β-actin 1:5000 (Sigma Aldrich; UK); anti-FOXO1 1:1000; anti-pFOXO1 1:500; anti-CPT1A 1:1000 (Cell Signaling Technology; UK)) followed by incubation with HRP-conjugated secondary antibodies (1:10,000 in 0.2% BSA; 1 h (ThermoFisher Scientific; UK). Proteins were detected by enhanced chemiluminescence (ECL) using Western Lightning plus ECL reagent (Perkin-Elmer; UK). Band densities were semi-quantified by densitometry performed using ImageJ software and normalised to β-actin loading control.

### Immunocytochemistry

Cells were fixed in 4% PFA (10–15 min) and permeabilised in 0.1% Triton-X in PBS-Tween (15 min). Cells were incubated in blocking buffer (3% BSA/1% goat serum; 40 min) prior to incubation with primary antibody (1% BSA/1% serum in PBS-Tween) at optimised concentrations (anti-SIRT1 1:250 (MERK Millipore; UK); anti-acetylated-FOXO1 1:100 (Santa Cruz Biotechnology; Germany); anti-CPT1A 1:300 (Cell Signaling Technology; UK)). Primary antibodies were detected with Alexa-Fluor 488 or Alexa-Fluor 594 secondary antibodies (1:1000 (ThermoFisher Scientific; UK)). Samples were then incubated with DAPI (1:5000 in TBS-Tween) for 5 min or coverslips were mounted using Fluoroshield DAPI-containing mounting medium (Sigma Aldrich; UK). Images were collected using either a DM4000B upright microscope (filter cubes A4 & L5 (Leica Microsystems)) or a Leica DMIRB inverted microscope (40x objective) controlled through Axiovision software version 4.8.2 (Carl Zeiss Ltd; UK). Change in fluorescence intensity was measured in pixels using ImageJ software.

### Metabolic analysis

Confluent HUVEC on gelatin-coated 24-well plates (for monolayer experiments) or HUVEC seeded onto Geltrex^TM^-coated 24-well plates at 25,000 cells/cm^2^ (for tube formation experiments) were incubated with or without compounds of interest for 4 or 5 h. Glycolytic rate was determined by measuring the production of ^3^H_2_O from 5–^3^H-glucose (17 MBq/mmol), separated using Dowex-1-Borate chromatography^57^. Change in the rate of FAO was determined by the production of ^3^H_2_O from 9,10–^3^H-palmitate (final specific activity 0.0185 MBq/ml), measured following separation of ^3^H_2_O by chloroform:methanol Folch extraction^58^. Glucose oxidation was measured by ^14^CO_2_-capture using D-U-^14^C-glucose (Perkin-Elmer; UK) at a final specific activity of 0.00925 MBq/ml^59^. Radioactivity was determined in technical duplicate using a Tri-carb 2800TR Liquid Scintillation Analyser and results calculated as nmol of substrate/minute/10^5^ cells.

Estimated ATP production was calculated from the rate of glycolysis, glucose oxidation and FAO. It was assumed that 2 moles of ATP were generated per mole of glucose that underwent glycolysis, 31 moles of ATP generated per mole of glucose oxidised and 106 moles of ATP generated per mole of fatty acid (palmitate) oxidised. Relative cellular ATP content was measured in EC monolayers using the CellTitre-Glo 2.0 assay kit from Promega, according to the manufacturer’s instructions.

Cellular oxygen consumption rate (OCR) was measured in EC monolayers using the Seahorse XFp extracellular flux analyzer (Agilent Technologies, UK). Baseline OCR was measured over a 30 min period with readings taken at 5 min intervals. GW0742 (100 nM) or medium alone was automatically injected into the appropriate wells and OCR was measured at 15 min intervals up to 4 h post-treatment.

### Mitochondrial activity

Overall mitochondrial activity was assessed by 3-(4,5 dimethylthiazol-2-yl)-2,5-diphenyltetrazolium bromide (MTT) reduction assay. After treatment, cells were incubated for 2 h (37 °C/5% CO_2_) with MTT solution (1 mg/ml in PBS) and the resultant formazan product was solubilised using solubilisation solution (DMSO; 37 °C; 20 min). Solubilisation solution was collected and transferred to a clear 96-well plate (50 µl/well in technical triplicate) prior to measurement of absorbance at 550 nm. Readings were corrected for background and results are expressed as a percentage of control.

### Statistical analysis

All data are expressed as means ± standard error of means (S.E.M) and unless stated otherwise, are from at least 3 independent experiments performed on separate EC isolates. Data analysis was performed using GraphPad Prism 6 software. Student’s t-test was employed for establishing significant differences between two groups. For the comparison of three or more groups analysis of variance (ANOVA) was applied followed by Bonferroni or Tukey post-hoc analysis as appropriate. In all cases, *p* < 0.05 was used to indicate statistical significance.

## Supplementary information

Supplementary Information.
