# Supplementary material for: Context-dependent regulation of endothelial cell metabolism: differential effects of the PPARβ/δ agonist GW0742 and VEGF-A
Source: Sci Rep. 2020 May 12;10:7849. doi: 10.1038/s41598-020-63900-0 (PMC7217938; doi:10.1038/s41598-020-63900-0)
Supplement: Supplementary file 1 — Supplementary Information. [file 41598_2020_63900_MOESM1_ESM.pdf]

Context-dependent regulation of endothelial cell metabolism: differential effects of the PPARβ/δ agonist GW0742 and VEGF-A

Ashton Faulkner[1, 2], Eleanor Lynam[1], Robert Purcell[1], Coleen Jones[1], Colleen Lopez[3], Mary Board[3], Kay-Dietrich Wagner[4], Nicole Wagner[4], Carolyn Carr[3] and Caroline Wheeler-Jones[1]*

[1] Department of Comparative Biomedical Sciences, Royal Veterinary College, London, UK

[2] Current address: Bristol Heart Institute, Bristol Medical School, University of Bristol, Bristol, UK

[3] Department of Physiology Anatomy & Genetics, University of Oxford, Oxford, UK

[4] Université Côte d'Azur, Institute of Biology Valrose, Nice (iBV), CNRS UMR7277, INSERM U1091, Nice, France.

Supplementary Methods

Additional tube formation analysis

For additional analysis of tube formation assays, phase-contrast images were analysed using the Angiogenesis analyser for ImageJ as previously described. Parameters measured included the average number of nodes and junctions together with the cumulative branch length.

Assessment of medium lactate concentrations

Lactate concentration in cell culture medium was determined using a colorimetric assay [1]. Briefly, the enzymes lactate oxidase (from Pediococcus sp.; 350 U/L final activity) and peroxidase (from horseradish; 2000 U/L final activity) were added to assay buffer (0.1M citric acid, 1mg/ml BSA, 0.1% $CaCl_2$, 0.02% sodium azide; adjusted to pH 6.0 with 1M Na_2HPO_4) in a 1:1:5 volume ratio with ABTS (0.015% final concentration), respectively. 200µl of complete assay buffer was combined with 5µl of either L-lactate standard (0-10mM) or sample medium per well of a 96-well plate in technical triplicate. Reactions were incubated in darkness for 30min at room temperature before absorbances were read at 405nm. After background correction results were normalised to total cellular protein and expressed as µg lactate/µg of protein.

Mitochondrial biogenesis

Since no single marker can determine mitochondrial biogenesis, evidence of mitochondrial biogenesis was assessed using a combination of markers. First, following extraction of total cellular DNA (DNeasy Blood & Tissue Kit; Qiagen; UK), a measure of the mitochondrial to genomic DNA ratio was performed by qPCR using primers directed against mitochondrial-encoded tRNA-Leu(UUR) and nuclear-encoded B2-microglobulin, as described by Rooney et al. (2015) [2]. Second, a change in mitochondrial transcription factor A (TFAM) gene expression (TaqMan^{TM}; Hs01082775_m1) was assessed by RT-qPCR; and third, the mitochondrial-specific stain, MitoTracker Green (ThermoFisher Scientific; UK), was used to visualise gross changes in mitochondrial number/morphology by confocal fluorescence microscopy. Following treatments, cells were incubated in experimental medium containing 200nM MitoTracker Green dye for 40min (37°C/5% CO_2). Confocal fluorescence images of mitochondria were obtained using a Leica SP5 confocal microscope through a 63x HCX PL APO lambda blue water immersion (NA=1.2) objective using Leica Application Suite advanced fluorescence software version 2.6 (Leica Microsystems).

Assessment of HUVEC proliferation by nuclear staining

HUVEC were seeded on gelatin-coated 24-well plates (30,000 cells/well) in experimental medium (supplemented with 10% FBS) containing compounds of interest (in triplicate). After 24h incubation the medium was aspirated, and cells washed in PBS before fixation in 4% paraformaldehyde (PFA) (10min). Cells were incubated in PBS containing DAPI nuclear stain (5min) prior to image capture (15 images/treatment group) using a Leica DMIRB inverted microscope (A4 filter cube; 10x objective (NA=0.25)). Nuclei were counted manually using ImageJ software.

Supplementary figures

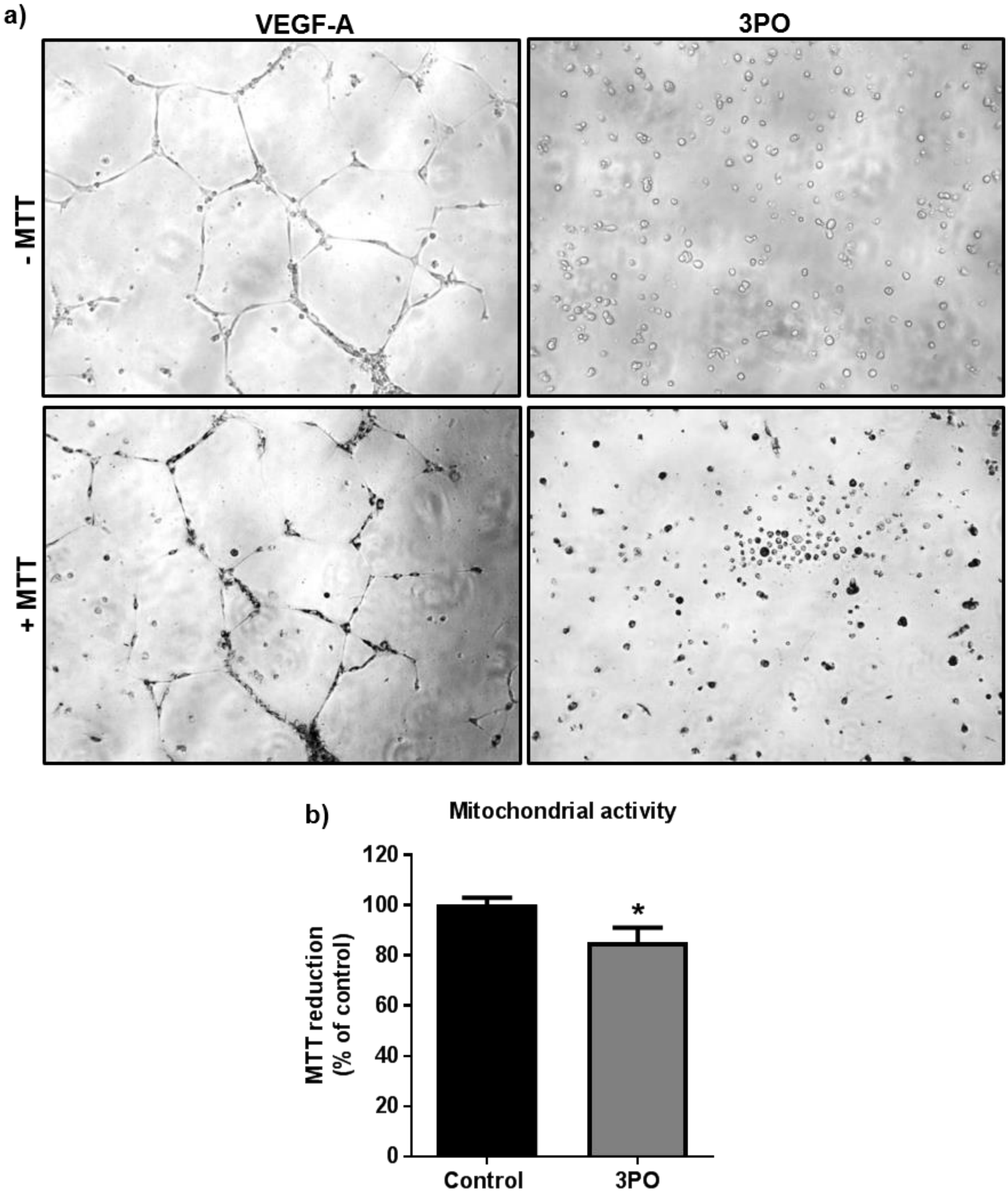

Supplementary fig. S1: Pharmacological inhibition of PFKFB3 causes a cytostatic effect on HUVEC. (a) Representative phase-contrast images (10x objective) showing 3PO (15µM; 16h) causing a cytostatic effect and VEGF-A (25ng/ml; 16h) promoting a dynamic effect on HUVEC when cultured on Geltrex. Under both conditions, mitochondria are still capable of reducing MTT. **(b)** MTT reduction as a measure of mitochondrial activity in HUVEC monolayers treated with or without 3PO (15µM) for 16h. Data represent means (± S.E.M) of n = 3 * p<0.05 *vs.* untreated control as determined by paired Student's *t*-test.

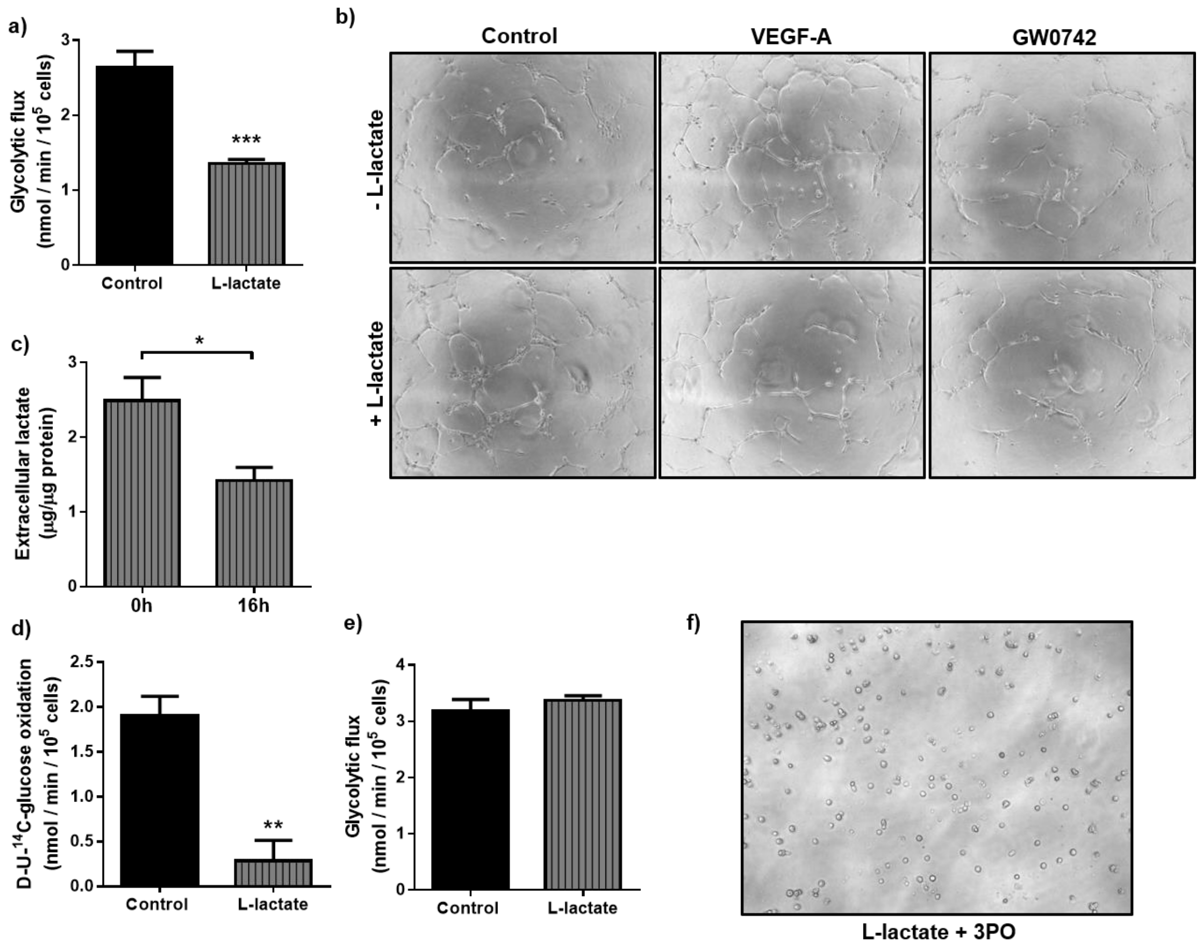

Supplementary fig. S2: L-lactate supplementation reduces glycolysis in HUVEC monolayers but not in HUVEC undergoing tubulogenesis. (a) Supplementing the culture medium with exogenous L-lactate (10mM; 4h) significantly reduces glycolytic flux in HUVEC monolayers. Data represent mean (± S.E.M) of *n* = 4 to 5 *** *p*<0.001 as determined by unpaired Student's *t*-test. **(b)** Representative phase-contrast images (10x objective) showing that L-lactate (10mM; 16h) exerts a pro-tubulogenic effect on HUVEC but its presence impairs VEGF-A- but not GW0742-induced tube-formation. **(c)** Medium lactate concentration is significantly reduced following tubulogenesis in the presence of L-lactate (10mM). **(d & e)** L-lactate (10mM; 4h) supplementation significantly reduces D-U-^{14}C-glucose oxidation but not glycolytic flux during tubulogenesis. Data represent mean (± S.E.M) of *n* = 4 to 5 ** *p*<0.01 *vs*. untreated control as determined by unpaired Student's *t*-test. **(f)** PFKFB3 inhibitor, 3PO (15µM) exerts a cytostatic effect on HUVEC in the presence of L-lactate (10mM) at 16h tubulogenesis.

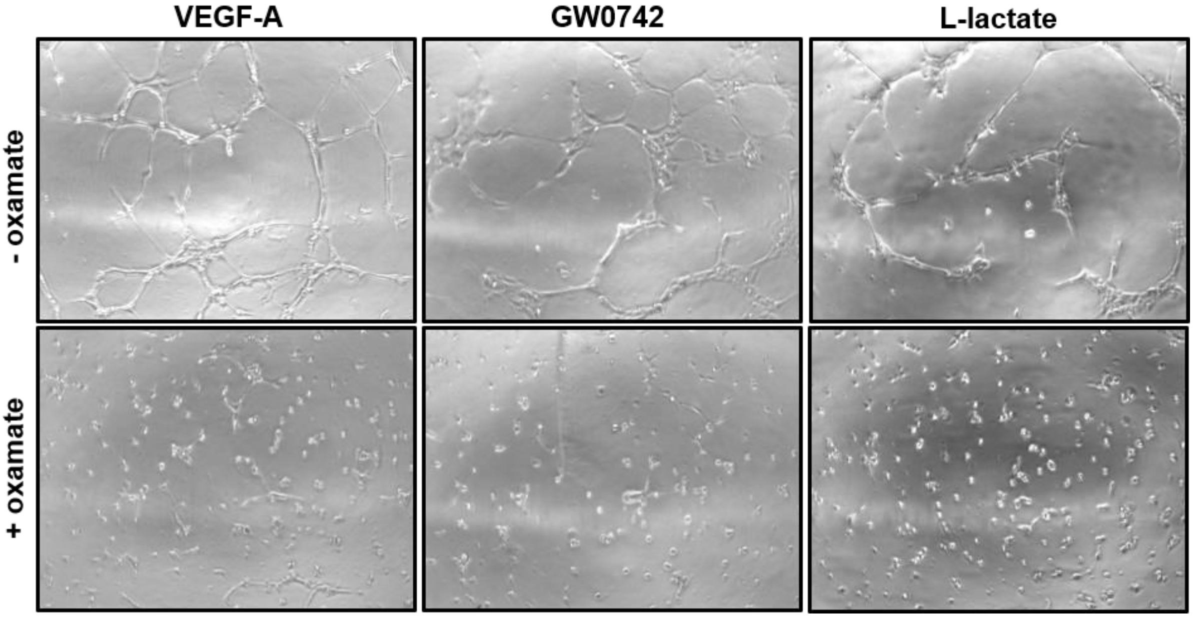

Supplementary fig. S3: An active LDH enzyme is essential for supporting HUVEC dynamic behaviour. Representative phase-contrast images (10x objective) showing the inhibitory effect of oxamate (1mM) on tubulogenesis induced by VEGF-A (25ng/ml), GW0742 (100nM) and L-lactate (10mM).

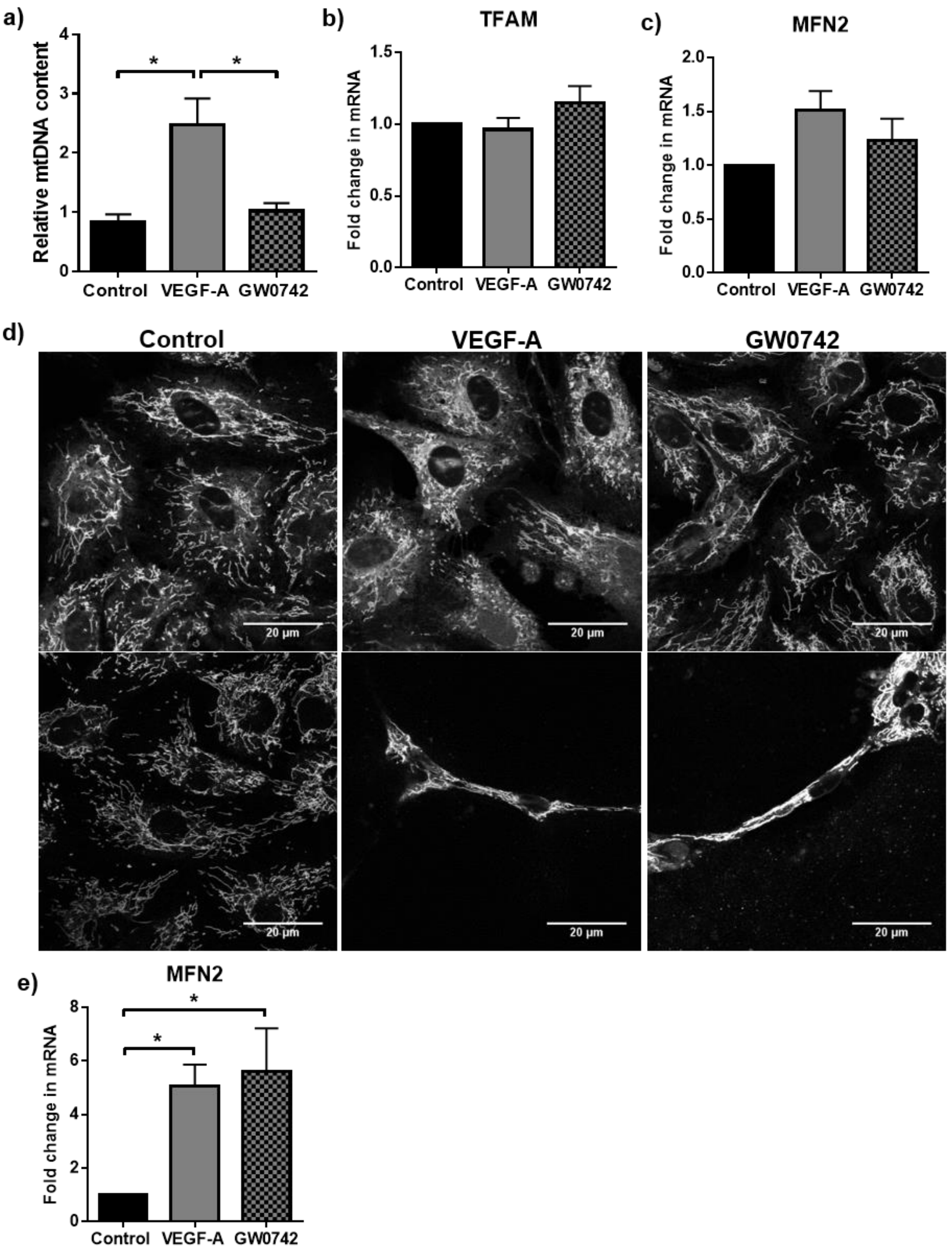

Supplementary fig. S4: Effect of VEGF-A and GW0742 on mitochondrial biogenesis and dynamics in confluent HUVEC monolayers and following tubulogenesis. (a) VEGF-A (25ng/ml; 4h) but not GW0742 (100nM; 4h) treatment significantly increases mitochondrial DNA content relative to untreated control in HUVEC monolayers. Data represent means (± S.E.M) of $n = 3$ * $p<0.05$ as determined by one-way ANOVA followed by Tukey's post-comparison test. **(b & c)** RT-qPCR showing that VEGF-A (25ng/ml; 4h) and GW0742 (100nM; 4h) treatment has no effect on TFAM or MFN2 mRNA expression in HUVEC monolayers. **(d)** Representative confocal immunofluorescence images (63x

objective) of the mitochondrial network (MitoTracker Green) in HUVEC monolayers under basal, VEGF-A (25ng/ml; 4h) and GW0742 (100nM; 4h) treated conditions and following transition from untreated monolayer to tube-like structures induced by either VEGF-A (25ng/ml; 16h) or GW0742 (100nM; 16h). *n* = 2 HUVEC isolates with both showing similar responses. **(e)** RT-qPCR showing that both VEGF-A (25ng/ml; 16h) and GW0742 (100nM; 16h) induced tubulogenesis is associated with significantly increased MFN2 mRNA expression. Data represent means (± S.E.M) of $n = 3$ * $p<0.05$ as determined by one-way ANOVA followed by Tukey's post-comparison test.

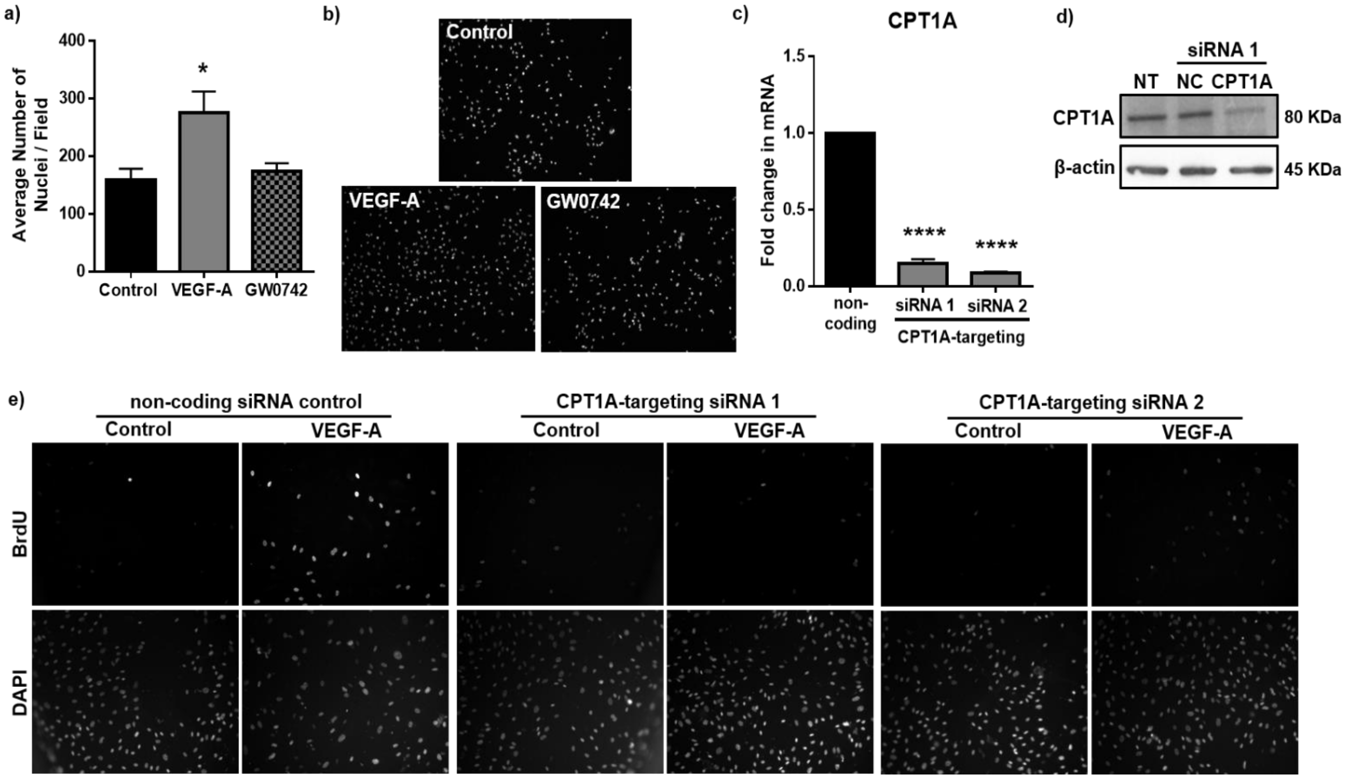

Supplementary fig. S5: VEGF-A-induced HUVEC proliferation is dependent on CPT1A. Quantification **(a)** and representative images (10x objective) **(b)** showing that VEGF-A (25ng/ml), but not GW0742 (100nM), induces HUVEC proliferation at 24h, as assessed by nuclear counting. Data represent means (± S.E.M) of n = 3 * p<0.05 *vs*. control as determined by one-way ANOVA followed by Bonferroni's post-comparison test. **(c)** RT-qPCR showing that both CPT1A-targeting siRNAs significantly reduce CPT1A mRNA expression in transfected HUVEC. Data represent means (± S.E.M) of n = 3 **** p<0.0001 *vs*. non-coding control as determined by one-way ANOVA followed by Bonferroni's post-comparison test. **(d)** Representative western blot showing a significant reduction in CPT1A expression 48h following transfection with CPT1A-targeting siRNA sequences (10nM) compared with non-transfected (NT) HUVEC and HUVEC transfected with non-coding (NC) sequences. **(e)** Representative images (20x objective) showing that VEGF-A-induced proliferation (BrdU assay) is reduced in CPT1A silenced HUVEC using two independent siRNAs.

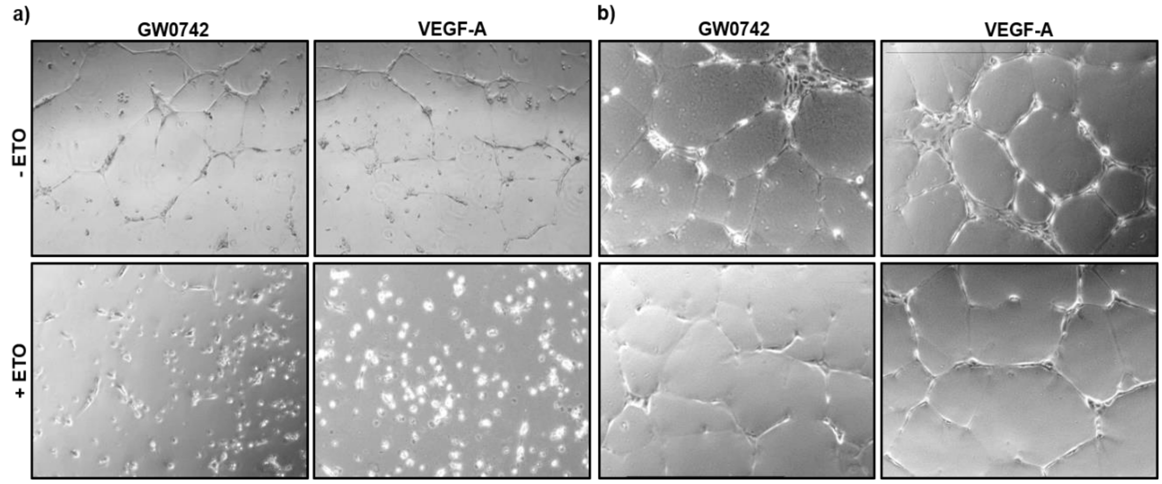

Supplementary fig. S6: The generic CPT1 inhibitor, etomoxir (ETO), has variable effects on HUVEC dynamic activity. Representative phase-contrast images (10x objective) of HUVEC induced to undergo tubulogenesis with either GW0742 (100nM) or VEGF-A (25ng/ml) for 16h in the absence and presence of ETO (1µM). Panel **a** shows a HUVEC isolate that responded negatively to the presence of ETO whilst panel **b** shows a HUVEC isolate that was still capable of forming tube-like structures in the presence of ETO.

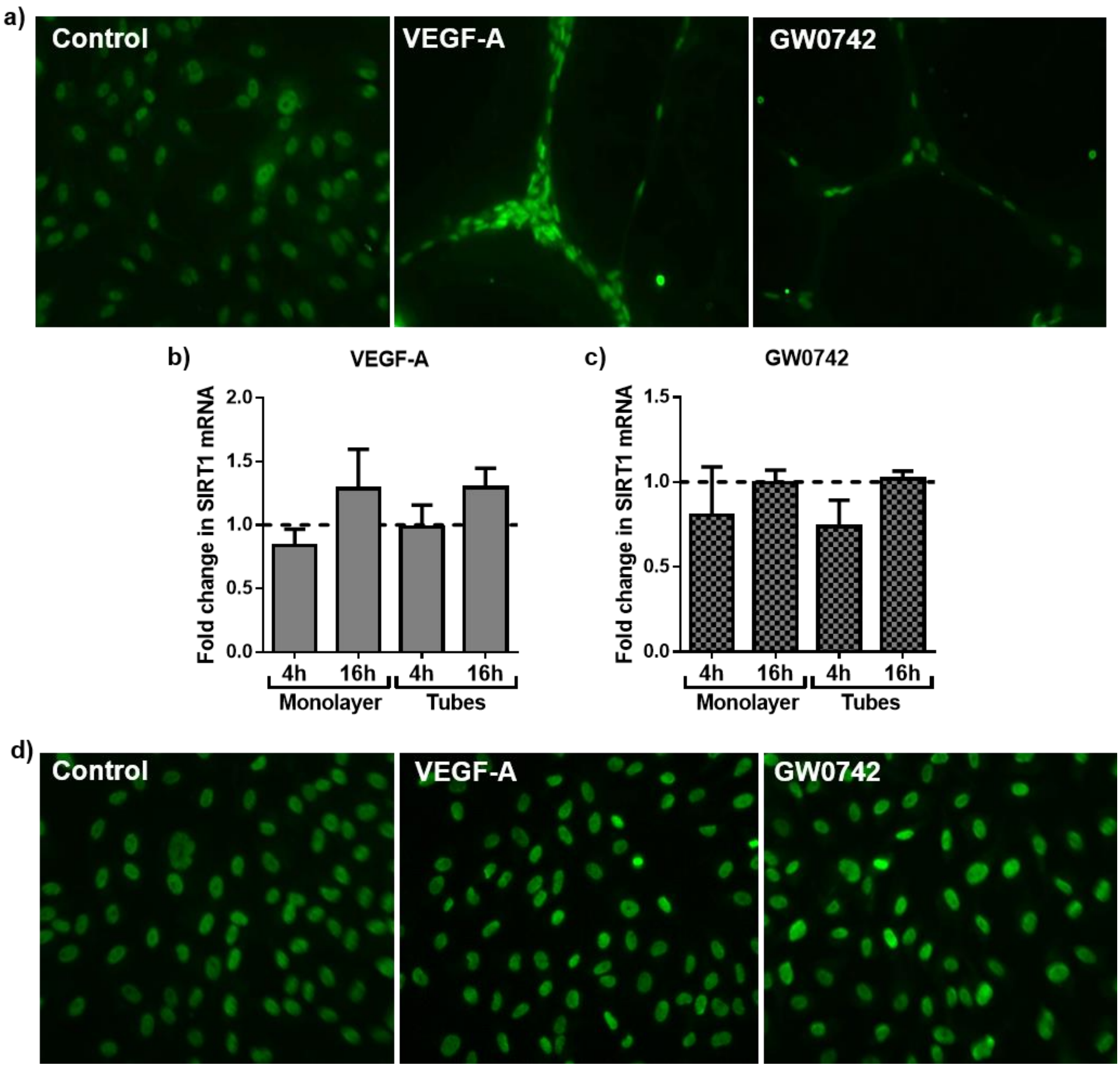

Supplementary fig. S7: SIRT1 retains a nuclear location in HUVEC and FOXO1 acetylation status remains unchanged under all treatment conditions. (a) Representative immunofluorescence images (40x objective) showing that SIRT1 (green) is predominantly located within the nuclear compartment of HUVEC monolayers and when induced to undergo tubulogenesis with either VEGF-A (25ng/ml) or GW0742 (100nM). **(b)** RT-qPCR showing no significant difference in SIRT1 mRNA expression at either 4h or 16h following VEGF-A (25ng/ml) treatment of HUVEC monolayers or tubes; *n* = 3 to 4 **(c)** RT-qPCR showing no significant difference in SIRT1 mRNA expression at either 4h or 16h following GW0742 (100nM) treatment of HUVEC monolayers or tubes; *n* = 3 to 4. **(d)** Representative immunofluorescence images (40x objective) showing that acetylated FOXO1 (green) is predominantly located within the nuclear compartment of HUVEC with levels remaining unchanged under all treatment conditions.

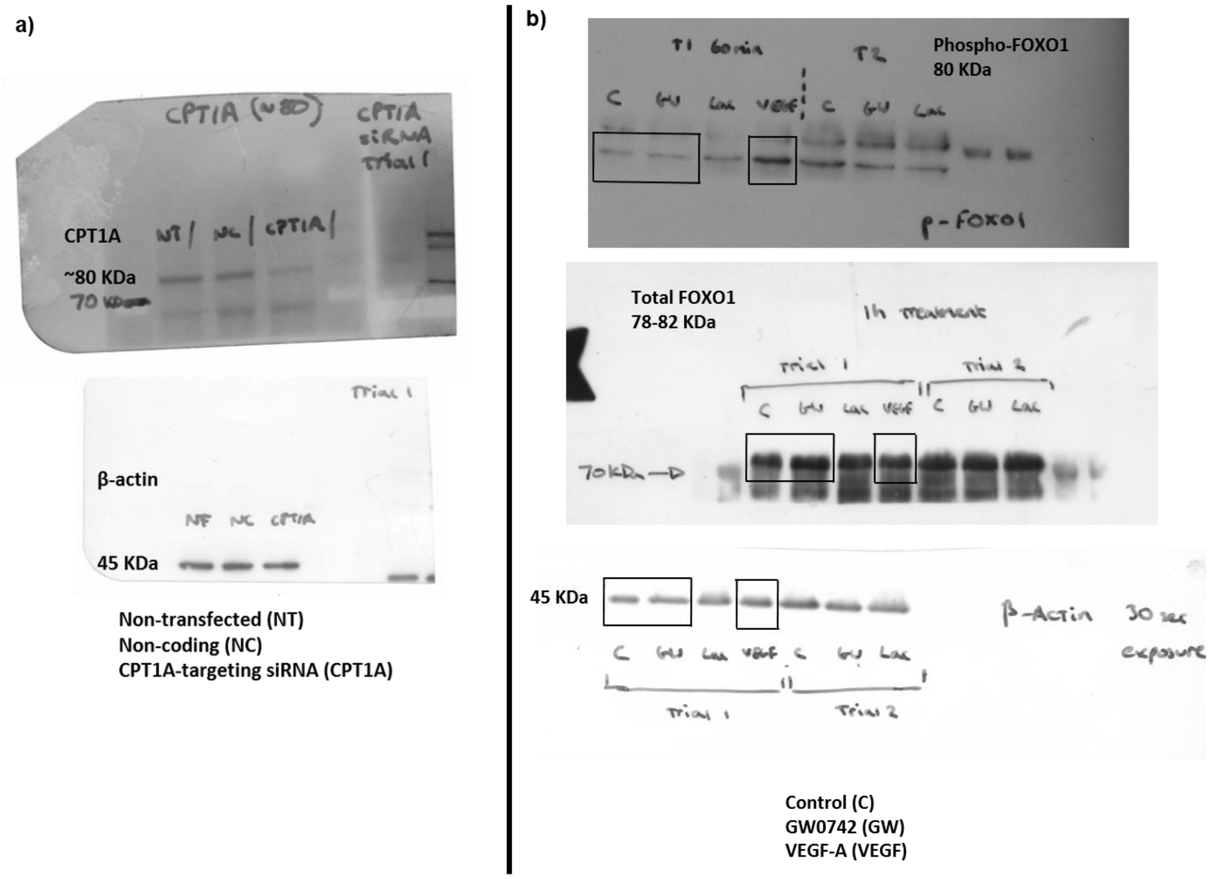

Supplementary fig. S8: Original non-edited western blots. (a) Western blots used for Supplementary fig. S5d showing successful silencing of CPT1A by siRNA and β-actin as loading control. **(b)** Western blots used for figure 6d showing the effect of GW0742 and VEGF-A on FOXO1 phosphorylation. Boxed area indicates bands used for final figure.

Supplementary tables

Supplementary Table S1: Primer sequences used for RT-qPCR

Target gene	Primer Sequence		NCBI Accession number / Reference
β-actin	Forward	GACAGGATGCAGAAGGAGATTACT	NM_001101
	Reverse	TGATCCACATCTGCTGGAAGGT	
CACT (SLC25A20)	Forward	GTGTCCAAGTGGATTGAGCA	NM_000387
	Reverse	TACACCCTGGGCTTTCTCAC	
LDHA	Forward	TTGAAGGGAGAGATGATGGA	NM_005566
	Reverse	CCAGCCGTGATAATGACCAG	
LDHB	Forward	TGCTCTTGTGGATGTTTTGG	NM_001315537
	Reverse	CTCTCCCCTTCTTGCTGACG	
MCT1	Forward	GTGGCTCAGCTCCGTATTGT	NM_003051
	Reverse	GAGCCGACCTAAAAGTGGTG	
MFN2	Forward	CCCCCTTGTCTTTATGCTGATGTT	[3]
	Reverse	TTTTGGGAGAGGTGTTGCTTATTTC	
SIRT1	Forward	CGTCTTATCCTCAGTTCTTGTG	NM_012238
	Reverse	ATCTCCATCAGTCCCAAATCC	
CPT1A	Forward	TCCAGTTGGCTTATCGTGGTG	NM_001876
	Reverse	TCCAGAGTCCGATTGATTTTTGC	
TBP	Forward	GGAGAGTTCTGGGATTGTAC	[4]
	Reverse	CTTATCCTCATGATTACCGCAG	

Supplementary Table S2: Additional tube-formation analysis relating to figure 2b showing the effects of GSK0660 on VEGF-A and GW0742-induced tubulogenesis

	Nodes (Mean ± SEM)	Junctions (Mean ± SEM)	Cumulative branch length (pixels) (Mean ± SEM)
**Control**	111.12 ± 25.78	31.88 ± 7.41	5331.27 ± 1052.97
**VEGF-A**	186.05 ± 46.24**	52.89 ± 12.92**	8086.11 ± 1400.44***
**GW0742**	148.36 ± 33.55*	42.83 ± 9.31*	6912.19 ± 1279.79**
**GSK0660**	117.89 ± 30.70	33.14 ± 8.53	5265.81 ± 1227.35
**VEGF-A + GSK0660**	153.47 ± 33.37	44.53 ± 9.84 **&**	7743.17 ± 1441.16 **&&&**
**GW0742 + GSK0660**	119.03 ± 29.36	34.29 ± 8.51	5698.02 ± 1265.14 **#**

***** $p<0.05$ vs. Control; ** $p<0.01$ vs. Control; *** $p<0.001$ vs. Control; # $p<0.05$ vs. GW0742 alone; **&** $p<0.05$ vs. GSK0660 alone; **&&&** $p<0.001$ vs. GSK0660 alone, as determined by repeated measures two-way ANOVA followed by Fisher's LSD test; n=6 independent HUVEC isolates.

Supplementary Table S3: Additional tube-formation analysis relating to figure 4c showing the effects of oligomycin on VEGF-A and GW0742-induced tubulogenesis

	Nodes (Mean ± SEM)	Junctions (Mean ± SEM)	Cumulative branch length (pixels) (Mean ± SEM)
**Control**	58.33 ± 10.95	16.56 ± 3.68	3078.50 ± 588.33
**VEGF-A**	89.44 ± 16.70*	25.78 ± 5.05*	4506.89 ± 817.10*
**GW0742**	82.74 ± 17.37*	23.69 ± 5.17*	4300.40 ± 946.80*
**Oligomycin**	80.89 ± 14.15*	22.72 ± 4.08*	4139.89 ± 873.40*
**VEGF-A + oligomycin**	78.77 ± 22.32	22.46 ± 6.37	4130.99 ± 849.56
**GW0742 + oligomycin**	58.56 ± 14.07 **#**	17.56 ± 4.14 **#**	3365.78 ± 755.34 **#**

* $p<0.05$ vs. Control; # $p<0.05$ vs. GW0742 alone, as determined by repeated measures two-way ANOVA followed by Fisher's LSD test; n=3 independent HUVEC isolates.

Supplementary Table S4: Additional tube-formation analysis relating to figure 4d showing the effects of L-lactate on VEGF-A and GW0742-induced tubulogenesis

	Nodes (Mean ± SEM)	Junctions (Mean ± SEM)	Cumulative branch length (pixels) (Mean ± SEM)
**Control**	111.12 ± 25.78	31.88 ± 7.41	5331.27 ± 1052.97
**VEGF-A**	186.06 ± 46.24**	52.89 ± 12.92**	8086.11 ± 1400.44**
**GW0742**	148.36 ± 33.55	42.83 ± 9.31	6912.19 ± 1279.79*
**Lactate**	122.74 ± 22.85	35.08 ± 6.70	6266.95 ± 1052.12
**VEGF-A + Lactate**	131.64 ± 18.64 #	38.20 ± 5.41 #	6675.64 ± 955.78 #
**GW0742 + Lactate**	111.36 ± 22.78	31.49 ± 6.44	5578.33 ± 1093.01 #

***** $p<0.05$ vs. Control; ** $p<0.01$ vs. Control; # $p<0.05$ vs. agonist alone, as determined by repeated measures two-way ANOVA followed by Fisher's LSD test; $n=6$ independent HUVEC isolates.

Supplementary Table S5: Additional tube-formation analysis relating to figure 6a showing the effects of EX-527 on VEGF-A and GW0742-induced tubulogenesis

	Nodes (Mean ± SEM)	Junctions (Mean ± SEM)	Cumulative branch length (pixels) (Mean ± SEM)
**Control**	48.39 ± 4.58	14.22 ± 1.80	2687.61 ± 381.85
**VEGF-A**	93.11 ± 10.44**	25.78 ± 2.93**	4083.67 ± 434.99*
**GW0742**	73.72 ± 11.23*	20.89 ± 3.62	3974.67 ± 567.34*
**EX-527**	43.50 ± 5.87	12.89 ± 1.27	2451.83 ± 173.94
**VEGF-A + EX-527**	61.11 ± 8.93 #	17.61 ± 2.67 #	3276.28 ± 523.54
**GW0742 + EX-527**	28.56 ± 2.56 ##	8.22 ± 0.70 ##	1709.78 ± 119.65 ##

***** $p<0.05$ vs. Control; ** $p<0.01$ vs. Control; # $p<0.05$ vs. VEGF-A alone; ## $p<0.01$ vs. GW0742 alone, as determined by repeated measures two-way ANOVA followed by Fisher's LSD test; $n=3$ independent HUVEC isolates.

Supplementary references

1 Lin, C.-Y., Chen, S.-H., Kou, G.-H. & Kuo, C.-M. An Enzymatic Microassay for Lactate Concentration in Blood and Hemolymph. *Acta Zoologica Taiwanica* **10**, 91-101 (1999).

2 Rooney, J. P. *et al.* PCR Based Determination of Mitochondrial DNA Copy Number in Multiple Species. *Methods in molecular biology* **1241**, 23-38 (2015).

3 Bach, D. *et al.* Expression of Mfn2, the Charcot-Marie-Tooth Neuropathy Type 2A Gene, in Human Skeletal Muscle : Effects of Type 2 Diabetes, Obesity, Weight Loss, and the Regulatory Role of Tumor Necrosis Factor α and Interleukin-6. *Diabetes* **54**, 2685-2693 (2005).

4 Goldberg, M. S. & Sharp, P. A. Pyruvate kinase M2-specific siRNA induces apoptosis and tumor regression. *The Journal of Experimental Medicine* **209**, 217-224 (2012).
